# Supplementary material for: Bivalent EGFR-Targeting DARPin-MMAE Conjugates
Source: Int J Mol Sci. 2022 Feb 23;23(5):2468. doi: 10.3390/ijms23052468 (PMC8909960; doi:10.3390/ijms23052468)
Supplement: Supplementary file 1 [file ijms-23-02468-s001.zip › ijms-1570299-supplementary.pdf]

Supplementary Information

Bivalent EGFR-targeting DARPin-MMAE conjugates

Lennard Karsten [1,+], Nils Janson [2,+], Vadim Le Joncour [3], Sarfaraz Alam [4], Benjamin Müller [5], Jayendrakishore Tanjore Ramanathan [3], Pirjo Laakkonen [3,6], Norbert Sewald [2,*] and Kristian M. Müller [1,*]

Contents

1 Details of DBCO-linker MMAE synthesis 2

2 Expression analyses of DARPin Dimer 2 3

3 MALDI-ToF mass spectrometry data 5

4 SDS-PAGE analyses 9

5 UV spectra and chromatograms 15

6 Cellular analyses 17

7 Animal data 20

8 LC-MS data 41

9 Chemicals, equipment and materials 45

10 Nucleic acid sequence of DARPin dimer 46

11 Amino acid sequences 47

11.1 hFGE 47

11.2 MtFGE 47

11.3 DARPin Monomer 48

11.4 DARPin Dimer (with C-terminal CTPSR) 48

11.5 DARPin Dimer (with N- and C-terminal CTPSR) 48

11.6 DARPin Dimer (with C-terminal CTPSR) 48

11.7 DARPinFc 48

11.8 scFv425 Fc 49

1 Details of DBCO-linker MMAE synthesis

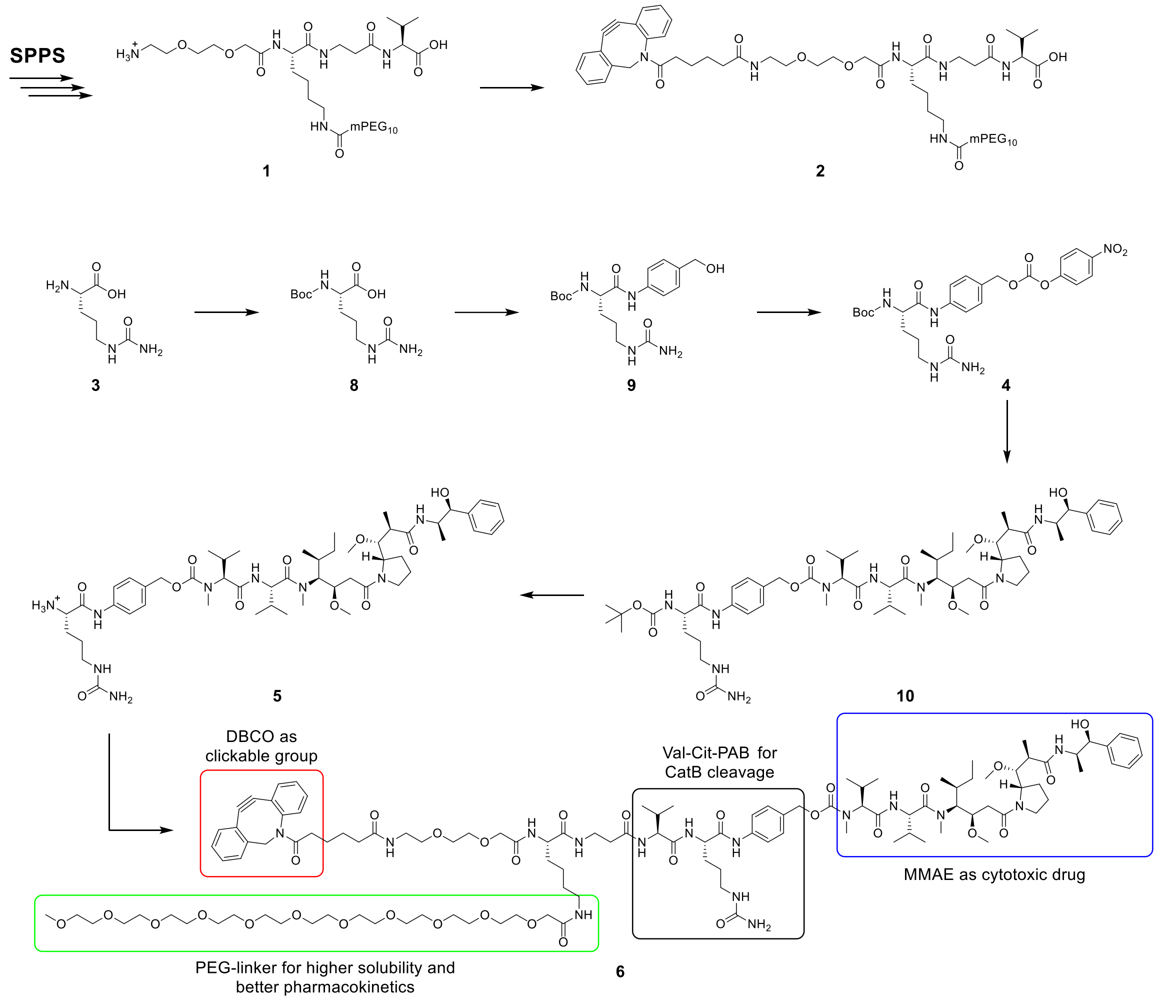

Fmoc-βAla-Val-Cit-OMe

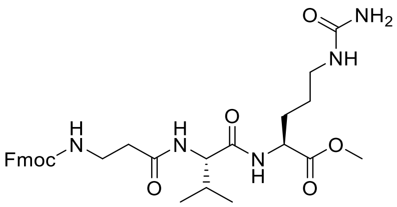

Procedure 1: H-Cit-OMe (5.96 mg, 31.5 mmol, 1 eq.), Fmoc-βAla-Val-OH (14.1 mg, 4.1 mmol, 1.3 eq.), HATU (14.3 mg, 37.8 mmol, 1.2 eq.) and HOAt (10.8 mg, 94.5 mmol, 3 eq.) were dissolved in DMF (500 µL) and DIPEA (13.8 µL, 94.5 mmol, 3 eq.) was added. The mixture was stirred at rt for 5 h and analyzed via LC-MS (~ 1% epimerization).

Procedure 2: H-Cit-OMe (5.96 mg, 31.5 mmol, 1 eq.), Fmoc-βAla-Val-OH (14.1 mg, 37.8 mmol, 1.3 eq.), PyAOP (17.91 mg, 41 mmol, 1.3 eq.) and HOAt (10.8 mg, 94.5 mmol, 3 eq.) were dissolved in DMF (500 µL) and DIPEA (13.8 µL, 94.5 mmol, 3 eq.) was added. The mixture was stirred at rt for 5 h and analyzed via LC-MS (< 1% epimerization).

Procedure 3: Fmoc-βAla-Val-OH (10.85 mg, 31.5 mmol, 1 eq.) and DIPEA (13.8 µL, 94.5 mmol, 3 eq.) were dissolved in DMF (500 µL) and the mixture was cooled to 0°C. Then, a solution of DEPBT (10.3 mg, 34.7 mmol, 1.1 eq.) in DMF (250 µL) and a solution H-Cit-OMe (5.96 mg, 31.5 mmol 1 eq.) in DMF (250 µL) of was added, respectively. The suspension was stirred at rt for 5 h and analyzed via LC-MS (> 20% epimerization).

2 Expression analyses of DARPin Dimer 2

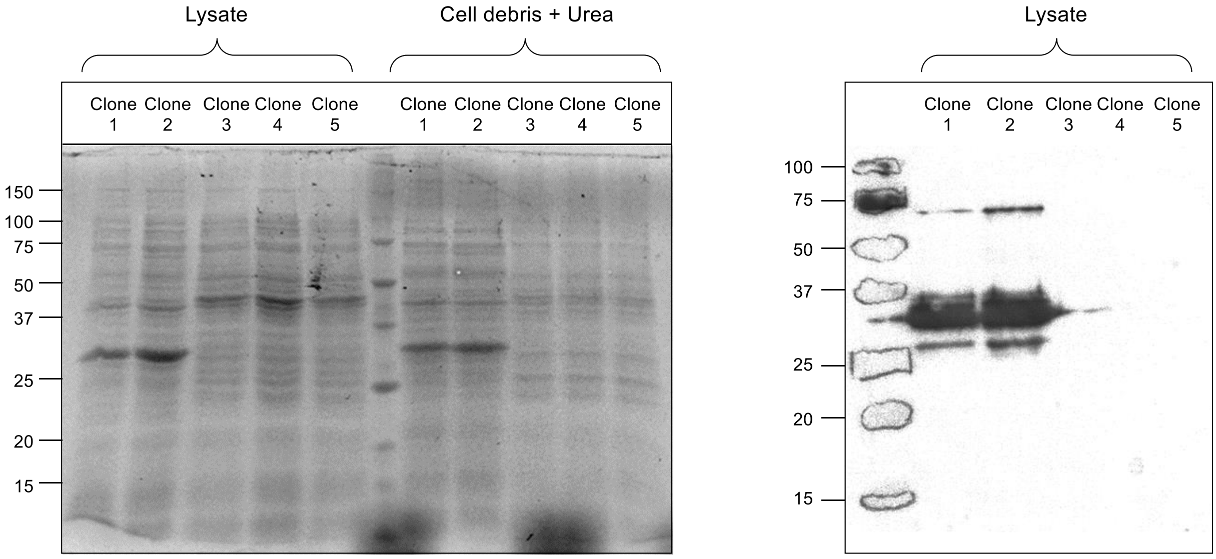

Figure S1: Expression control of 5 selected clones. Left: SDS-PAGE (12.5%) analysis with a 250 kDa-ladder protein marker of the DARPin Dimer (with N- and C-terminal CTPSR) (**DD2**) expression. Supernatants of lysate and urea-treated cell debris were analyzed. An intense band in clones 1 and 2 at approximately 34 kDa indicates overexpression of the desired construct. Right: Chemiluminescence image of the Western blot. The antibodies used were an anti-His-tag antibody and a peroxidase-containing coupled antibody with a chemiluminescence substrate and peroxide solution. A signal at around 34 kDa verified the successful over-expression of the desired His-tagged protein.

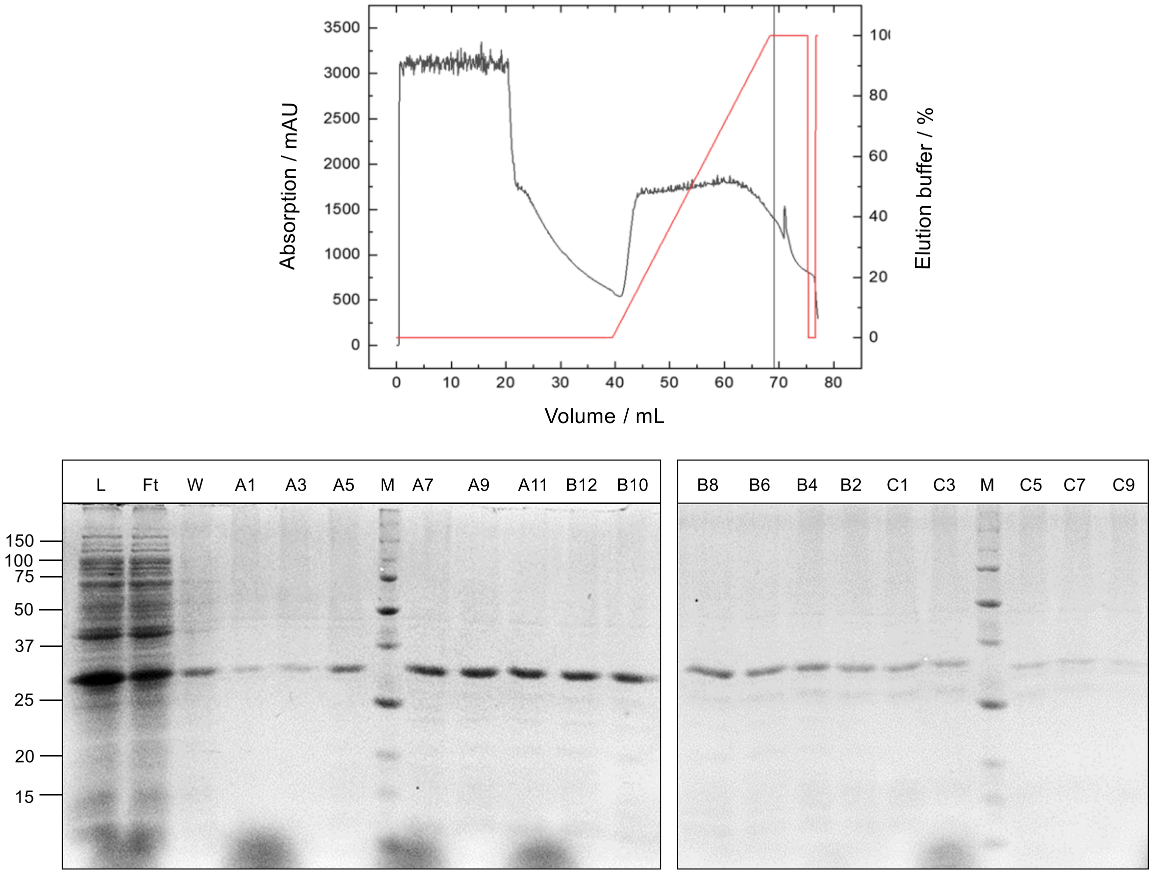

Figure S2: Purification of **DD2**. Top: Chromatogram of HisTrap (1 mL GE Healthcare). Binding buffer: 50 mM phosphate, 300 mL NaCl, 250 mM Imidazol, pH 8.0. Elution buffer: 50 mM phosphate, 300 mL NaCl, 250 mM imidazole, pH 8.0. Bottom: Coomassie-stained reducing SDS-PAGE (12.5%). L = Load, Ft = flowthrough, W = wash, M = molecular weight marker. The desired construct could be purified with high purity as demonstrated by SDS-PAGE.

3 MALDI-ToF mass spectrometry data

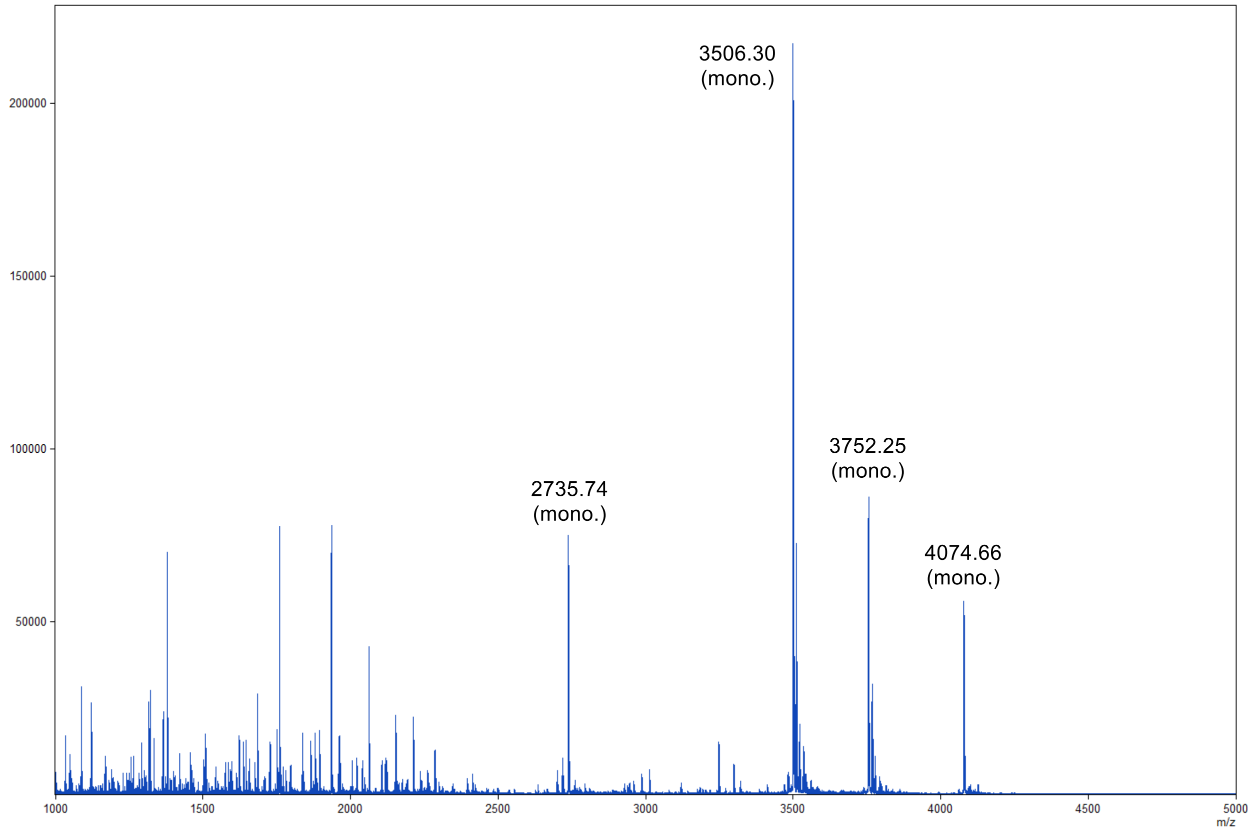

Figure S3: MALDI-ToF MS analysis of **DD2** after tryptic digest.

Table S1: Corresponding calculated and obtained *m/z* of **DD2**.

Peptide sequence	Position	m/z_{calc}	m/z_{found}
ILMANGADVNADDTWGWTPL HLAAYQGHLEIVEVLLK	37-70 and 207-243	4074.061 (mono.)	4074.66
TAFDISIDNGNEDLAEILQG GGGSGGGGSGGGGSGGGGSD LGK	149-191	3751.674 (mono.)	3752.25
NGADVNAYDYIGWTPLHLAA DGHLEIVEVLLK	71-102, 244-275, 103-135 and 276-308	3506.777 (mono.)	3506.30
TAFDISIDNGNEDLAEILQL CTPSR	322-346	2735.301 (mono.)	2735.74

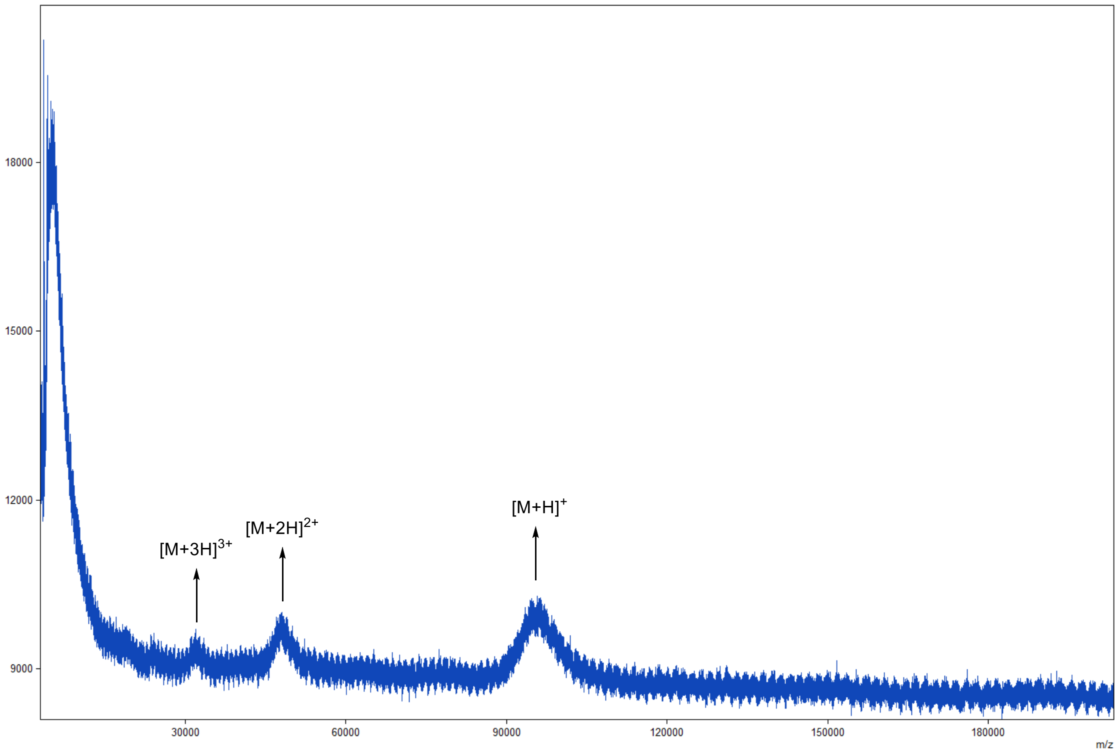

Figure S4: MALDI-ToF MS analysis of DARPinFc (whole protein).

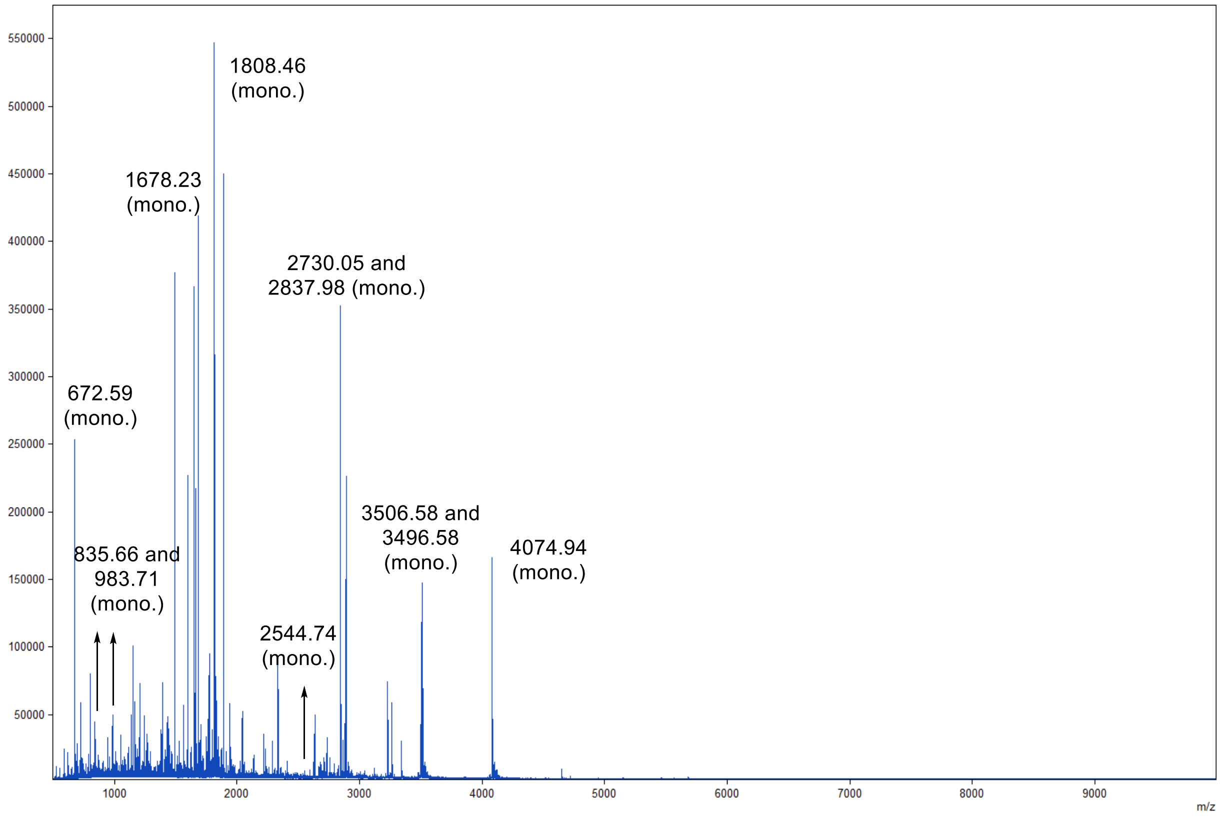

Figure S5: MALDI-ToF MS analysis of DARPinFc after tryptic digest.

Table S2: Corresponding calculated and obtained *m/z* of DARPinFc.

Peptide sequence	Position	m/z_{calc}	m/z_{found}
ILMANGADVNADDTWGWTPL HLAAYQGHLEIVEVLLK	21-57	4074.0792 (mono.)	4074.94
NGADVNAYDYIGWTPLHLAA DGHLEIVEVLLK	58-89	3506.7953 (mono.)	3506.30
NGADVNASDYIGDTPLHLAA HNGHLEIVEVLLK	90-122	3495.7866 (mono.)	3496.58
TAFDISIDNGNEDLAEILQG GSGGGSGDK	136-164	2837.3071 (mono.)	2837.98
THTCPPCPAPELLGGPSVFL FPPKPK	165-190	2730.4145 (mono.)	2730.05
GFYPSDIAVEWESNGQPENN YK	313-334	2544.1313 (mono.)	2544.74
VVSVLTVLHQDWLNGK	244-259	1808.0064 (mono.)	1808.46
FNWYVDGVEVHNAK	217-230	1677.8019 (mono.)	1678.23
AAHHHHHH	403-410	983.4455 (mono.)	983.71
DTLMISR	269-276	835.4342 (mono.)	835.66
LLEAAR	7-12	672.4039 (mono.)	672.59

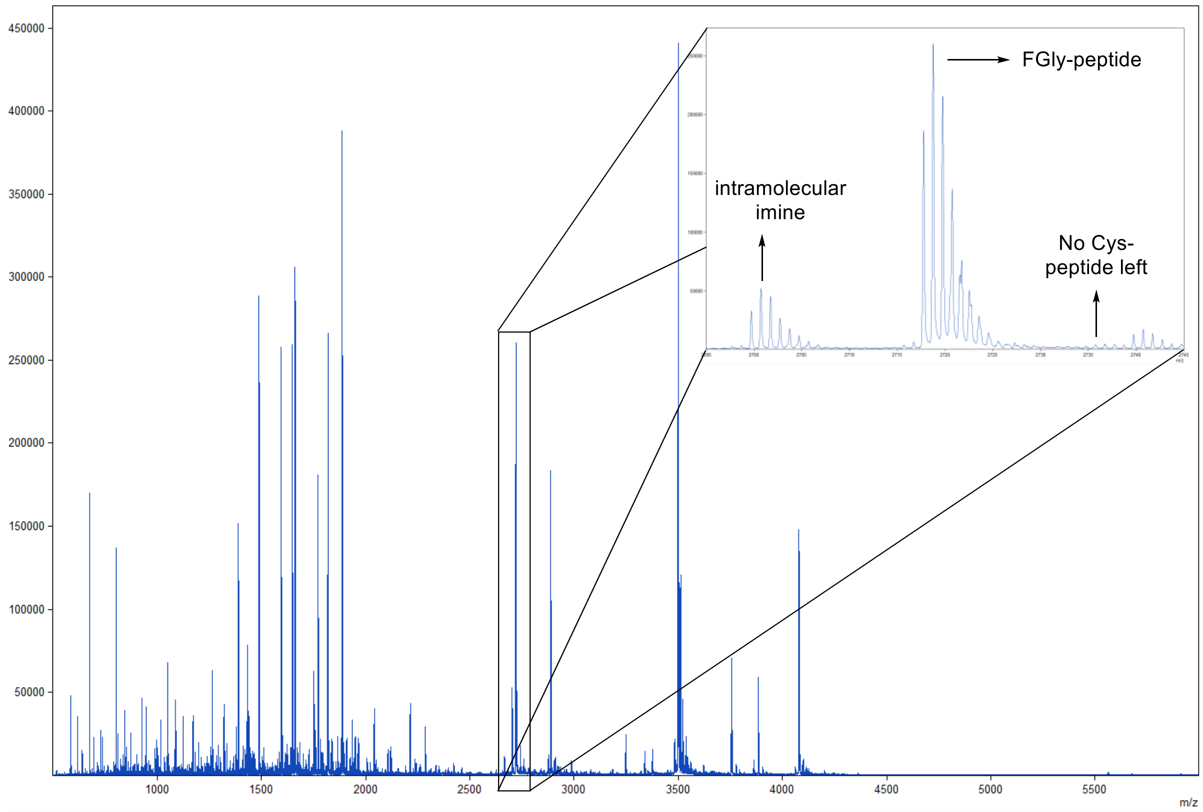

Figure S6: MALDI-ToF MS analysis of FGly-DARPin Dimer (**DD1**) using DNPH as matrix.

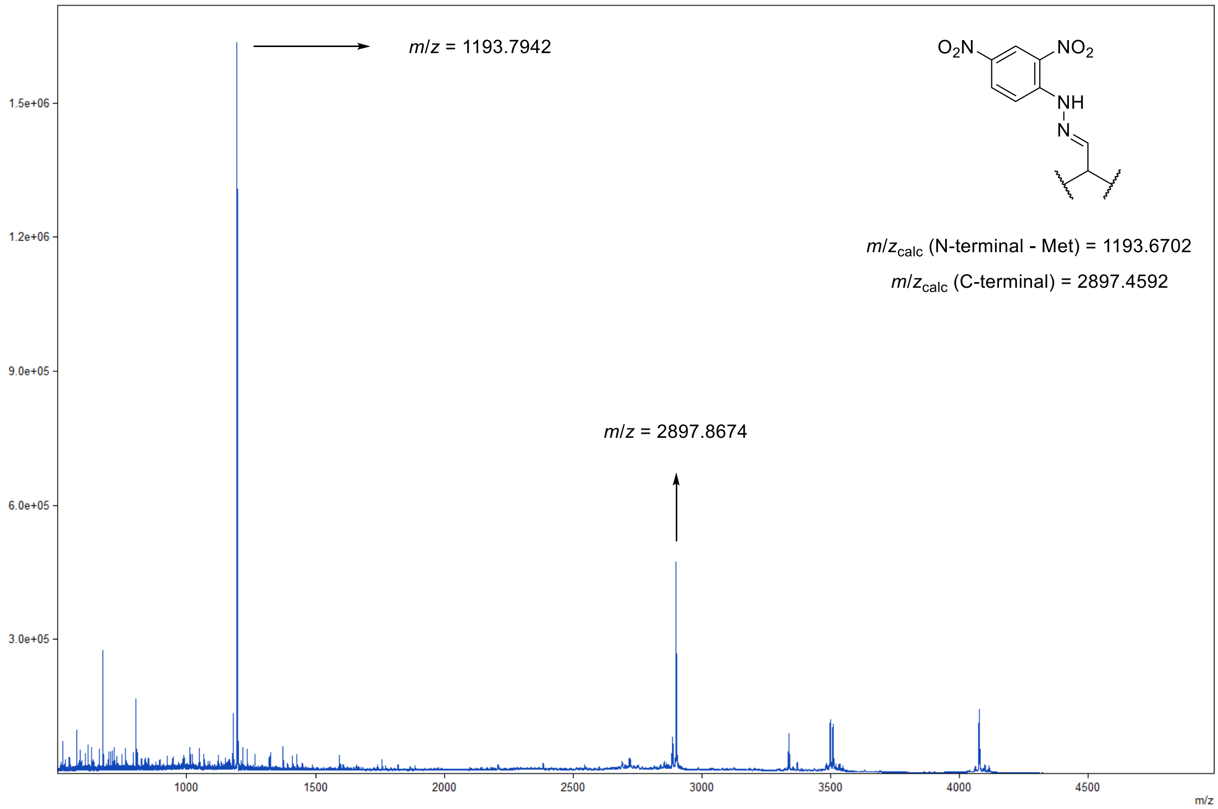

Figure S7: MALDI-ToF MS analysis of FGly-DARPin Dimer (**DD2**) using DNPH as matrix. CLCCA could not be used to detect the N-terminal aldehyde.

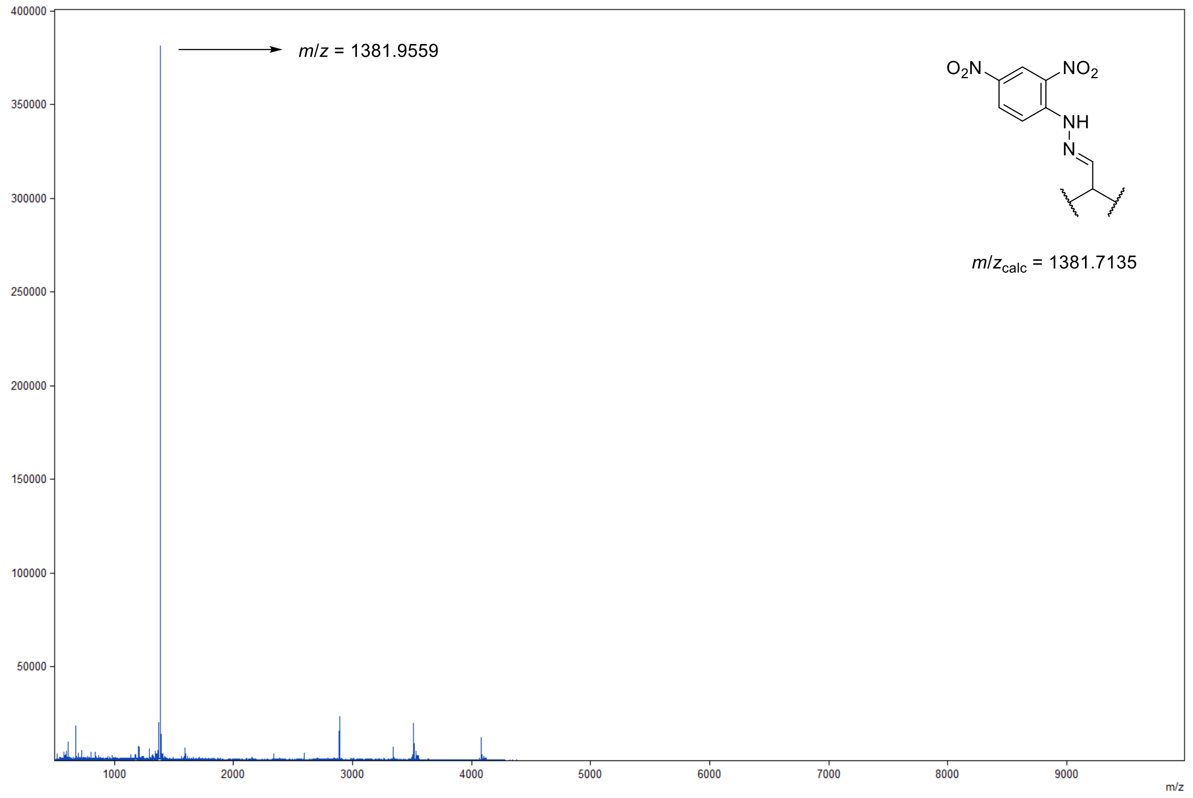

Figure S8: MALDI-ToF MS analysis of FGly-DARPinFc using DNPH as matrix. CLCCA could not be used to detect the aldehyde.

4 SDS-PAGE analyses

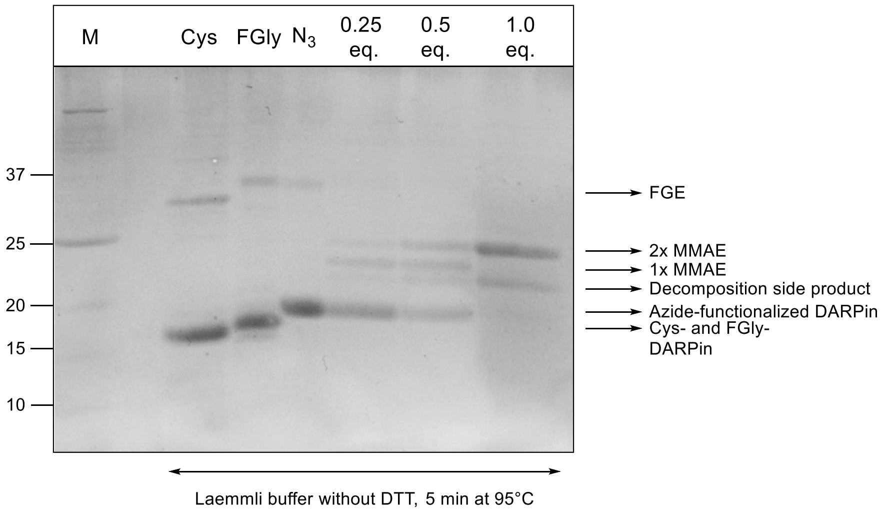

Figure S9: Coomassie-stained SDS-PAGE (15%) of DARPin Monomer (**DM**) (Cys) converted by FGE (FGly) and conjugated with *tandem* Knoevenagel-azide (N_3) and DBCO-PEG$_3$-Val-Cit-PAB-MMAE (0.25 – 1 eq.). M = molecular marker. In the reaction with 0.25 equivalents per azide, two bands can be observed at about 23 and 24 kDa, which can be assigned to the mono- and the double conjugate. Increasing the equivalents to one resulted in a complete conversion to the desired double conjugate. The formation of a band at about 22 kDa can be attributed to a heat-induced decomposition product (cleavage of the linker) during SDS preparation. Lowering the temperature resulted in the formation of a distinct band (See Fig. S10).

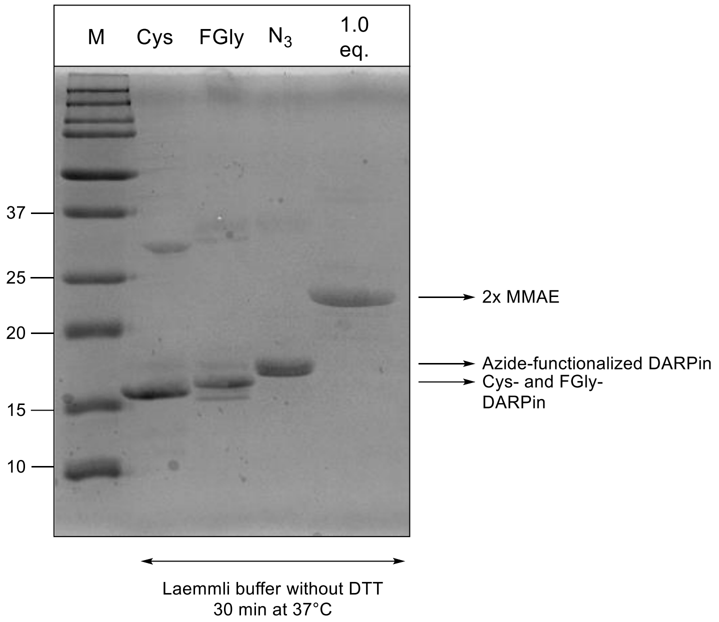

Figure S10: Coomassie-stained SDS-PAGE (15%) of **DM** (Cys) converted by FGE (FGly) and conjugated with *tandem* Knoevenagel-azide (N_3) and DBCO-PEG$_3$-Val-Cit-PAB-MMAE (1.0 eq.). M = molecular marker. The decomposition product (observed in Fig. S9) could be suppressed by lowering the temperature during the SDS-PAGE preparation. As a result, only one distinct band at approximately 24 kDa can be observed in the SDS gel, which can be assigned to the desired double MMAE conjugate.

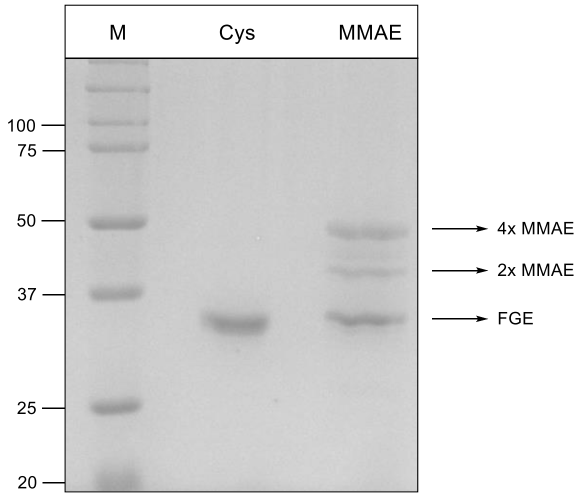

Figure S11: Coomassie-stained SDS-PAGE (15%) of **DD2** (Cys) converted by hFGE and conjugated with *tandem* Knoevenagel-azide (N_3) and DBCO-PEG$_3$-Val-Cit-PAB-MMAE (MMAE). M = molecular marker. The conju-gation did not proceed completely. A 2x (at ca. 40 kDa) to 4x MMAE (at ca. 48 kDa) conjugate ratio of ~20% to ~80% was achieved. Residual hFGE can be seen at 36 kDa. Increasing concentrations, equivalents, tem-peratures, and incubation times, as well as switching to MtFGE, did not result in complete conversion to the desired 4x MMAE conjugate (data not shown).

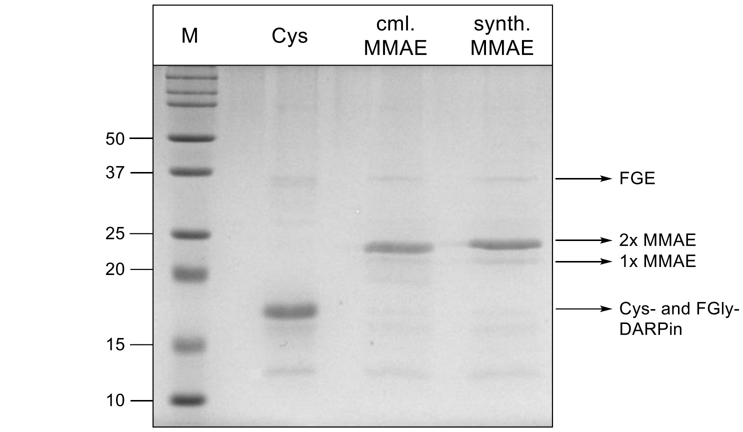

Figure S12: Coomassie-stained SDS-PAGE (15%) of **DM** (Cys) converted by FGE and conjugated with *tandem* Knoevenagel-azide and commercially DBCO-PEG$_3$-Val-Cit-PAB-MMAE (coml. MMAE) or DBCO-PEG$_2$-Lys(mPEG$_{10}$)-βAla-Val-Cit-PAB-MMAE (synth. MMAE). M = molecular marker. In both cases, a distinct band at ca. 24 kDa can be observed, indicating almost same conjugation efficiency for both, commercially and synthesized MMAE-linker.

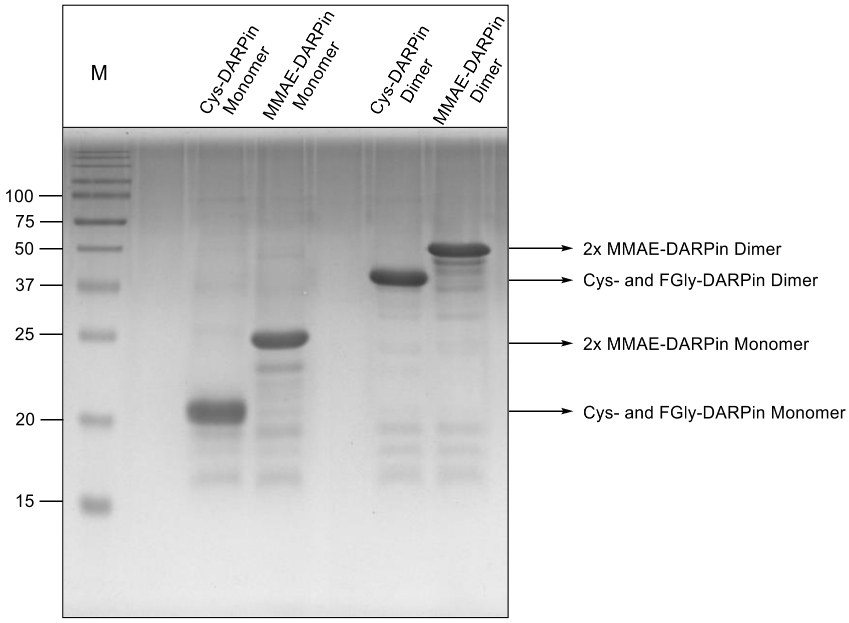

Figure S13: Coomassie-stained SDS-PAGE (15%) of **DM** and **DD1** converted by FGE and conjugated with *tan-dem* Knoevenagel-azide (N_3) and DBCO-PEG$_2$-Lys(mPEG$_{10}$)-βAla-Val-Cit-PAB-MMAE (MMAE). M = mo-lecular marker. In both approaches, a distinct band for the respective 2x MMAE conjugate was observed at approximately 24 kDa and 48 kDa, respectively. Faint other bands may be assigned to conjugates with lower protein to drug ratios or starting material, indicating almost complete conversion to the desired conjugates.

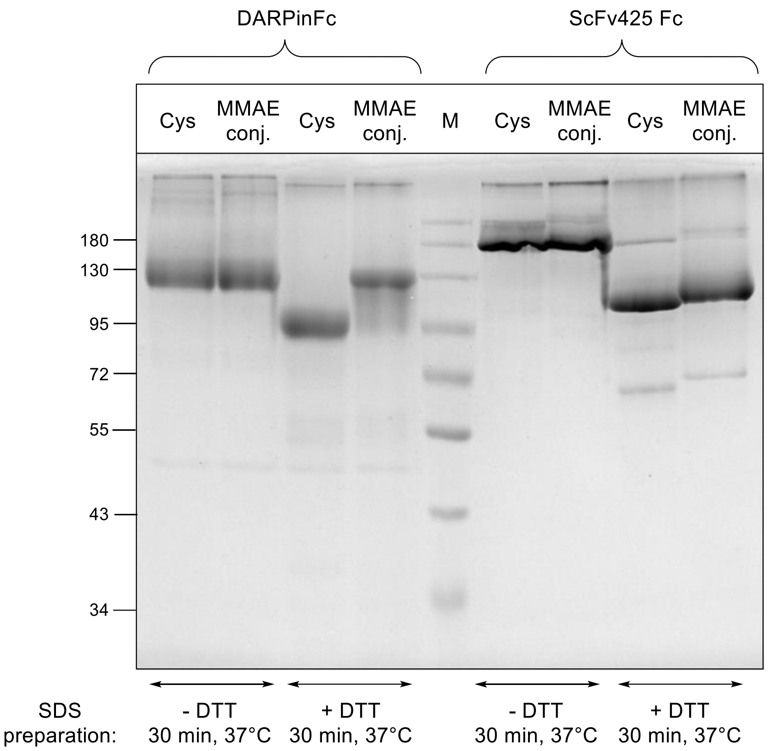

Figure S14: Reducing (5 mM) and non-reducing Coomassie-stained SDS-PAGE (10%) of DARPinFC (**DFc**) and **ScFv425 Fc** converted by FGE and conjugated with *tandem* Knoevenagel-azide and DBCO-PEG$_2$-Lys(mPEG$_{10}$)-βAla-Val-Cit-PAB-MMAE (MMAE). M = molecular marker. The samples without DTT run significantly higher than those with DTT. This is probably due to the fact that the easily accessible disulfides are reduced under the given reduction conditions (30 min at 37°C) in samples with DTT. This dissolves secondary/tertiary structures or leads to improved attachment of SDS. The disulfides of the hinge region are more stable under the given reduction conditions, leading to the observation of mainly the desired dimer rather than the monomers (45-55 kDa and 55-72 kDa). The complete conversion of the click reaction can be seen by the formation of a distinct, slightly upward-shifted band in the samples with DTT (MMAE conj.), confirming the high efficiency of the method we developed.

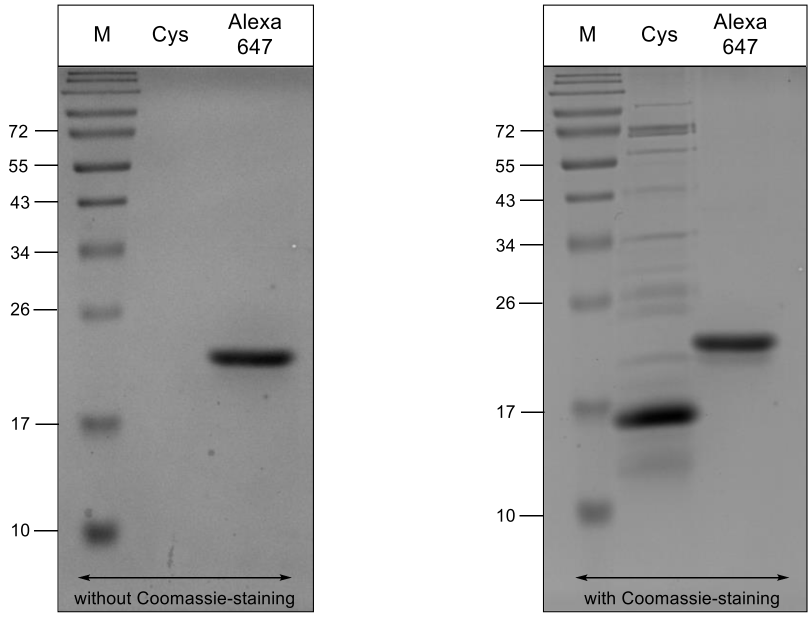

Figure S15: SDS PAGE analysis (with 5 mM DTT) of **DM** Alexa fluor 647 conjugate. Left: SDS gel (15%) before Coomassie staining. A pre-stained protein ladder was used, which is why the protein ladder can be seen before Coomassie staining. The staining of our desired protein conjugate at about 23 kDa is caused by the blue color of the fluorophore. A weak band can be observed directly underneath, indicating almost com-plete conversion to the doubly-modified conjugate. Right: SDS gel (15%) after Coomassie staining. The SDS gel confirms the successful purification of the conjugate and the twofold modification. Furthermore, all bac-terial proteins (which could not be separated with HisTrap) could be removed by HIC. Fluorophore-to-protein ratio of ~ 1.5 – 2.

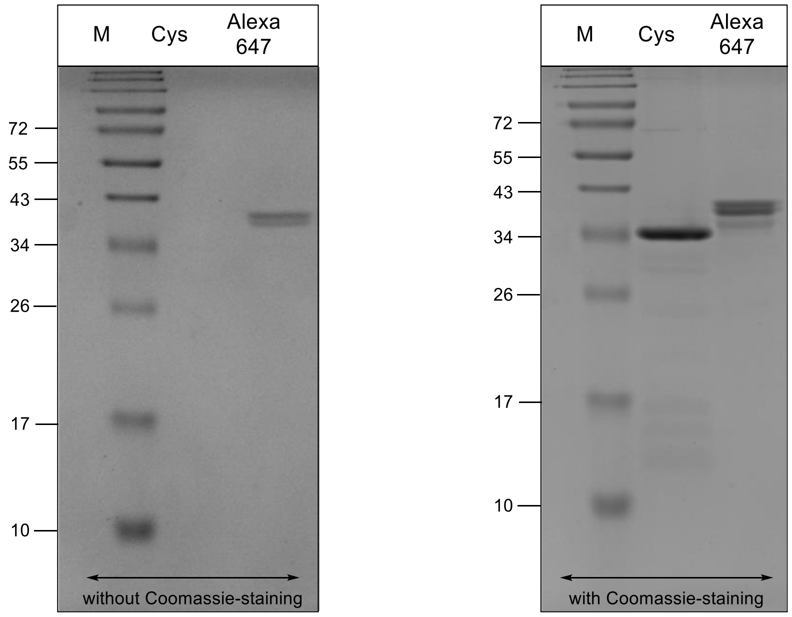

Figure S16: SDS PAGE analysis (with 5 mM DTT) of **DD1** Alexa fluor 647 conjugate. Left: SDS gel (15%) before Coomassie staining. A pre-stained protein ladder was used. The staining of the conjugate is caused by the blue color of the fluorophore. Two bands are observed in the SDS gel, indicating that the protein was ap-proximately 50% mono-modified and 50% double-modified. Right: SDS gel (15%) after Coomassie staining. The SDS gel confirms the successful purification of the conjugate. In addition to the mono and double con-jugate, a faint band could also be observed directly underneath, which can be assigned to the azide-modified DARPin. However, the protein could be purified from all bacterial proteins (which could not be separated with HisTrap) by HIC. Fluorophore-to-protein ratio of ~ 1 – 1.5.

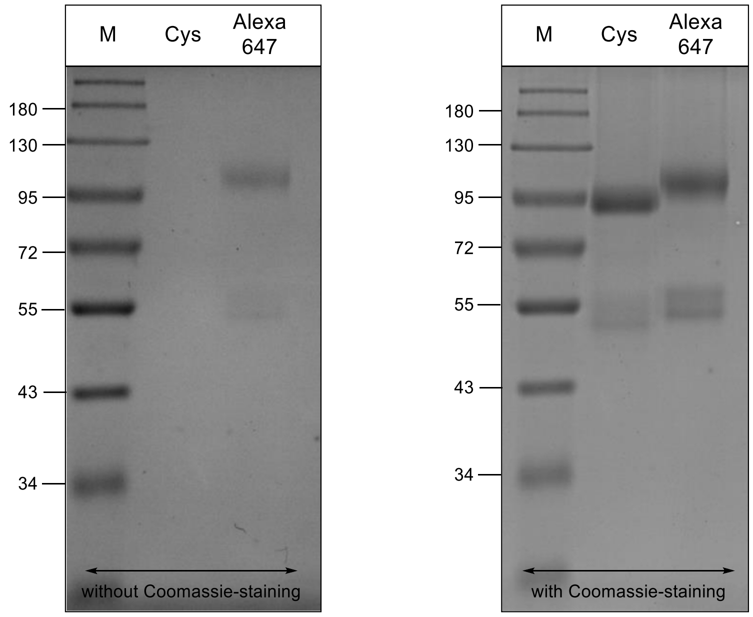

Figure S17: SDS PAGE analysis (with 5 mM DTT) of **DFc** Alexa fluor 647 conjugate. Left: SDS gel (10%) before Coomassie staining. A pre-stained protein ladder was used. The staining of the conjugate is caused by the blue color of the fluorophore. A distinct band at about 105 kDa shows successful fluorescent labelling of the construct with Alexa fluor 647. Two bands at about 52 and 57 kDa (monomer bands) indicate that the DARPinFc construct has been both, mono- and double modified per FGly. Right: SDS gel (10%) after Coo-massie staining. The SDS gel confirms the successful modification. This can be seen by a band shifted to higher molecular weights (95 kDa vs 105 kDa). The absence of the unmodified monomer band at about 50 kDa shows that each FGly was modified at least once with Alexa Fluor 647. Fluorophore-to-protein ration of ~ 2 – 3.

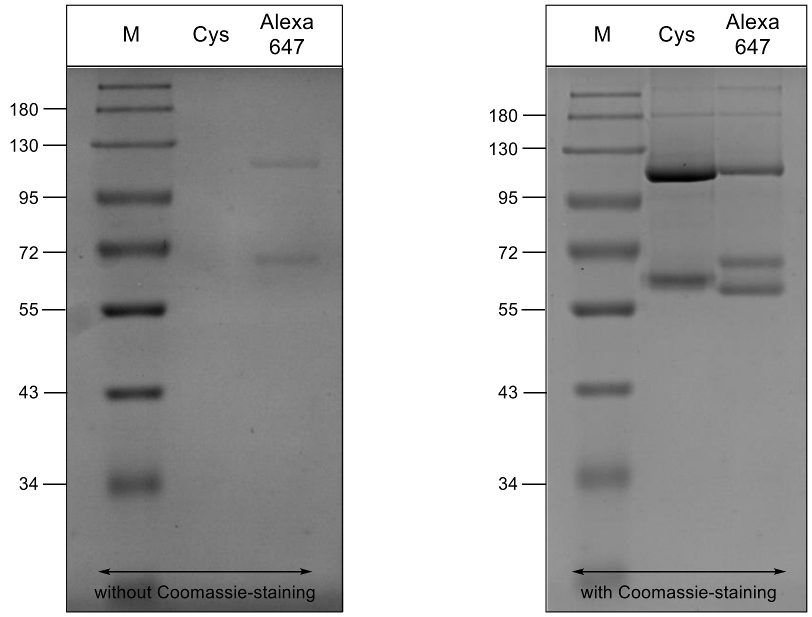

Figure S18: SDS PAGE analysis (with 5 mM DTT) of **ScFv425 Fc** Alexa fluor 647 conjugate. Left: SDS gel (10%) before Coomassie staining. A pre-stained protein ladder was used. The staining of the conjugate is caused by the blue color of the fluorophore. Two distinct bands at ca. 120 kDa (desired dimer) and 65 kDa (mono-mer) showing successful fluorescence labeling of the protein. Right: SDS gel (10%) after Coomassie staining. The SDS gel confirms the successful modification due to a slight shift to higher molecular weights. Interest-ingly, two bands (65 kDa and 60 kDa) are observed for the monomer, with the upper band successfully modified with Alexa Fluor 647. The second band is slightly lower than the actual monomer band (about 62

kDa) observed with the non-conjugated protein (Cys). This is due to the cleavage of the tag after formation of FGly. [35] Fluorophore-to-protein ration of ~ 1 – 2.

5 UV spectra and chromatograms

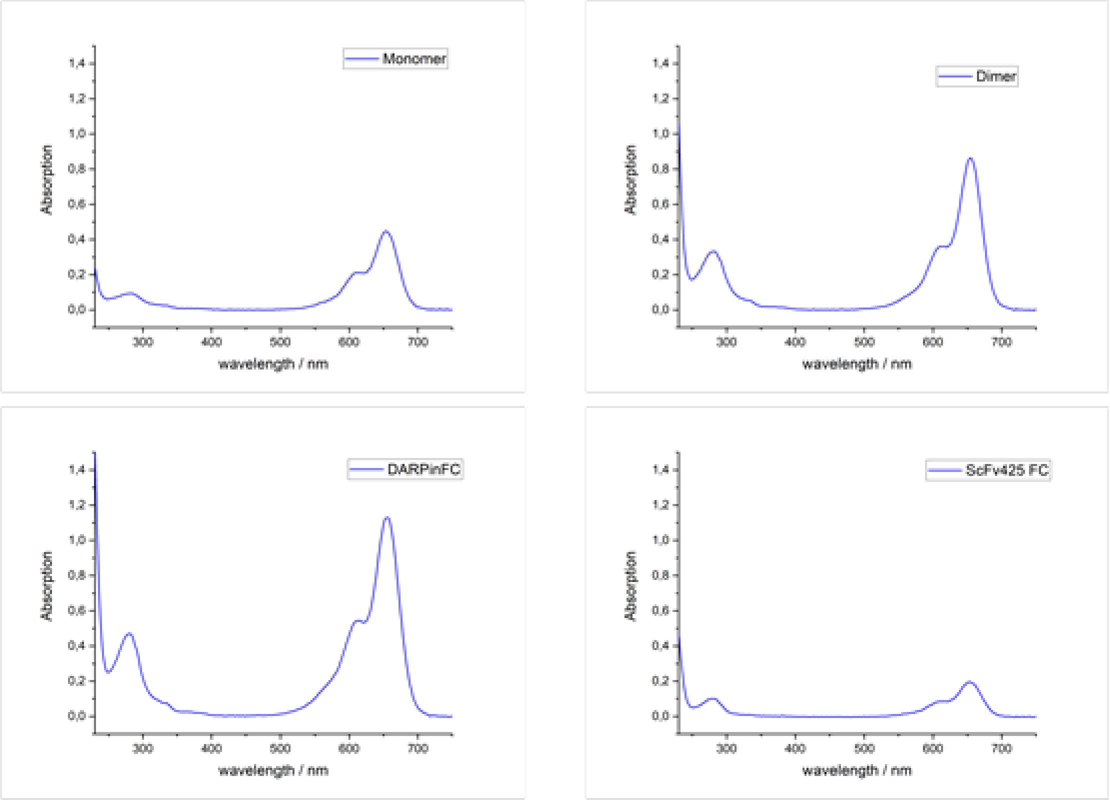

Figure S19: UV absorption spectra of the Alexa-647 conjugates. Shown is the absorption between 230 and 750 nm. Between 550 and 700 nm, the specific absorbance of the fluorophore (Alexa fluor 647) can be observed. The extinction coefficient (270.000 cm^{-1} M^{-1}) was used to determine the concentration of the bound fluoro-phore.

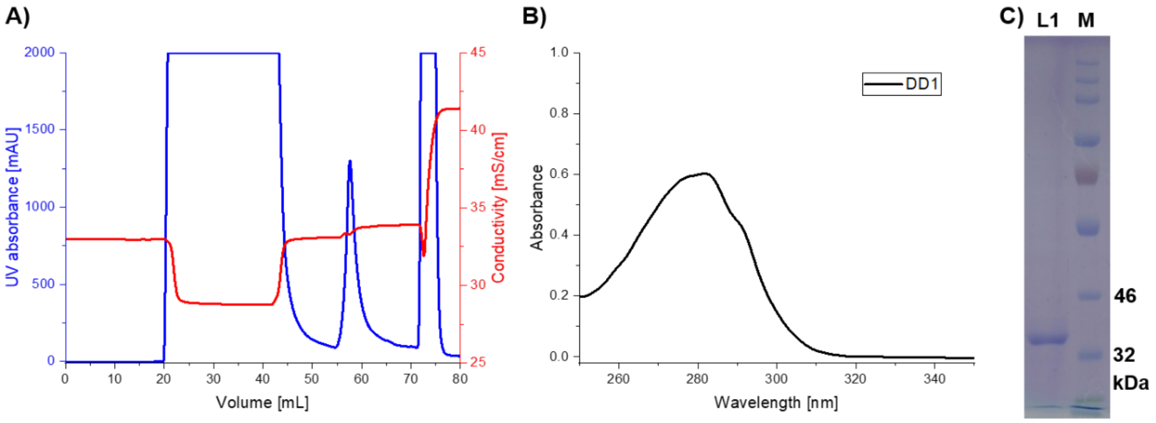

Figure S20: A) UV absorbance (blue) and conductivity (red) of IMAC (Ni-NTA) for **DD1** purification. **B)** UV absorbance spectra of purified **DD1**. **C)** Coomassie-stained SDS gel with **L1:** reduced **DD1**, M: molecular marker

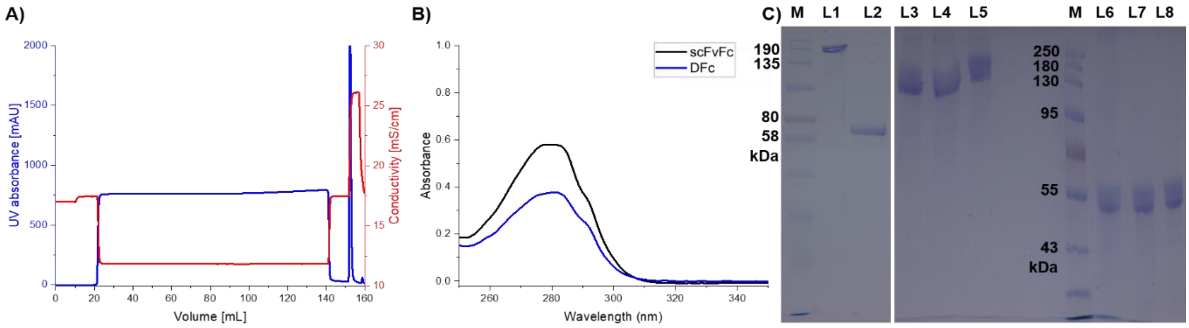

Figure S21: A) UV absorbance (blue) and conductivity (red) of protein A purification of **DFc**. **B)** UV absorbance spectra of purified **scFvFc** (black) and **DFc** (blue). **C)** Coomassie-stained SDS gels with **L1:** non-reduced **scFvFc**, **L2:** reduced **scFvFc**, **L3-L5:** non-reduced **DFc**, **L6-L8:** reduced **DFc**, M: molecular marker.

6 Cellular analyses

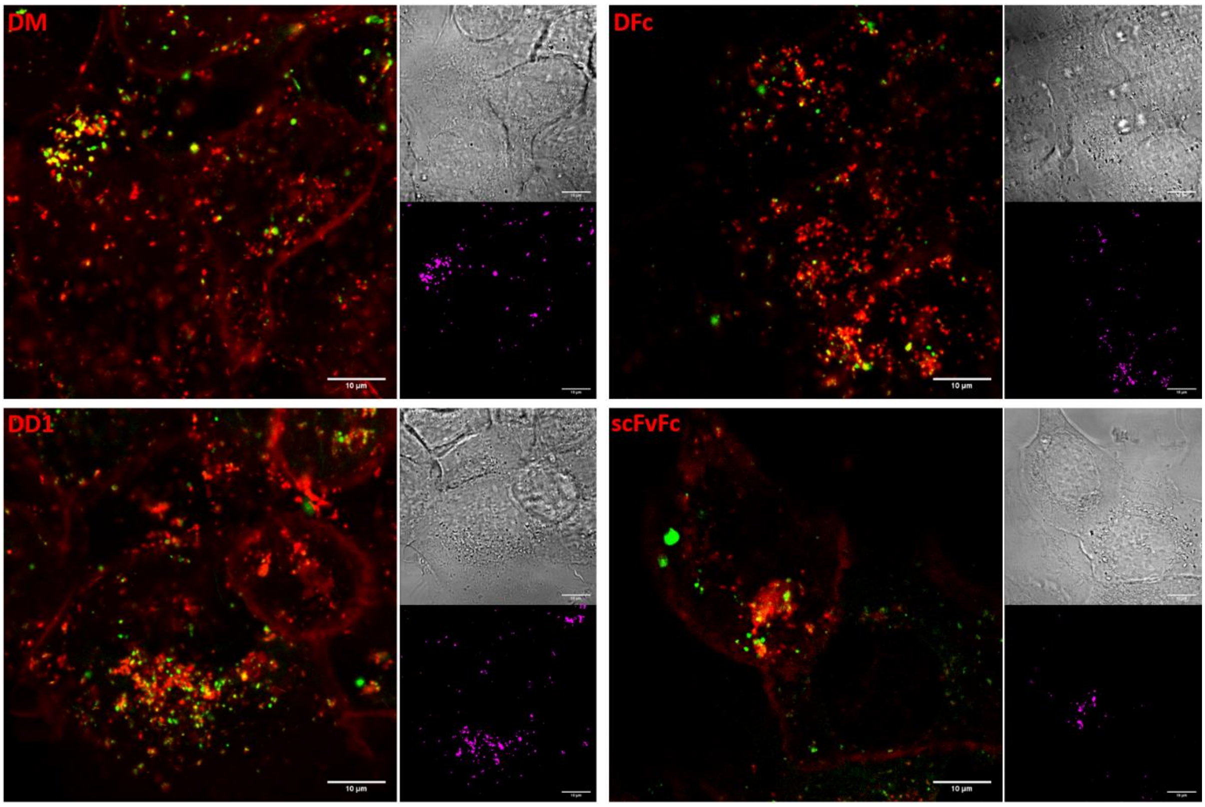

Figure S22: A431 cells expressing high level of EGFR were incubated with 100 nM Alexa Fluor 647-labeled **DM** (upper left), **DD1** (lower left), **DFc** (upper right) or **scFvFc** (lower right) dye-conjugates (red). Cells were incubated with dye-conjugates for 10 minutes, washed, and imaged after 6 hours at 37 °C. Lysosomes (green) were stained using LysoTracker Green DND-26. Miniaturized images on the right hand side show either colocalization (lower) of Alexa Fluor 647 signals with the lysosomes pseudocolored in magenta or bright-field images (upper).

Table S3. Colocalization analysis using the ImageJ plugin JACoP [1] with the Pearson's coefficient r and the Mandels coefficients M1 (DND-26 overlap AF647) and M2 (AF647 overlap DND-26).

Dye-conjugate		2 h 37°C	6 h 37°C
**DM**	r	0.281	0.431
	M1	0.339	0.721
	M2	0.260	0.239
**DD1**	r	0.211	0.407
	M1	0.490	0.816
	M2	0.075	0.165
**DFc**	r	0.592	0.410
	M1	0.610	0.736
	M2	0.120	0.110
**scFvFc**	r	0.258	0.201
	M1	0.371	0.204
	M2	0.038	0.102

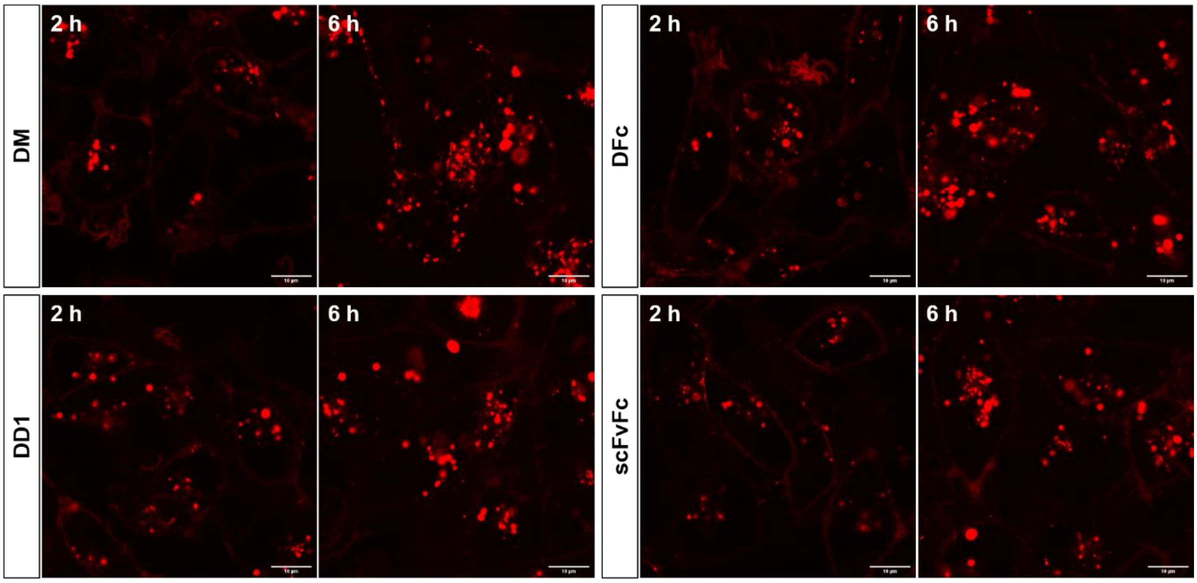

Figure S23: Breast cancer cells MDA-MB-231 with a tenfold lower EGFR level compared to A431 were incu-bated with 100 nM Alexa Fluor 647-labeled **DM** (upper left), **DD1** (lower left), **DFc** (upper right) or **scFvFc** (lower right) dye-conjugates (red). Cells were incubated with dye-conjugates for 30 minutes, washed, and imaged after 2 or 6 hours at 37 °C.

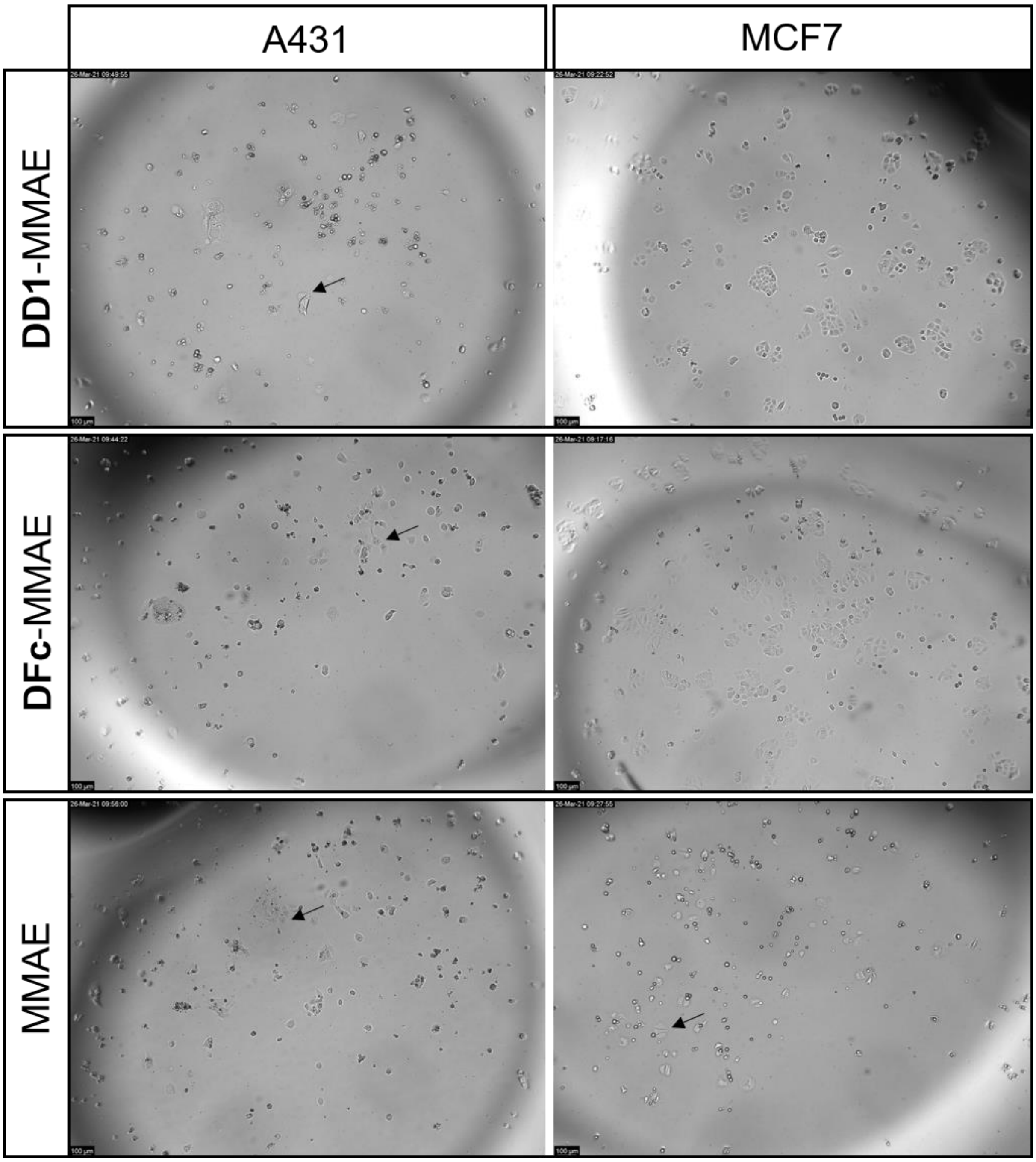

Figure S24: In vitro cytotoxicity of the anti-EGFR DARPin-MMAE conjugates. 1×10^3 adherent A431 or MCF7 cells were seeded in 96-well plates and incubated with **DD1**-MMAE, **DFc**-MMAE or free MMAE at a final concentration of 5 nM in cell culture medium for 72 h at 37°C and 5 % CO_2-atmosphere in a final volume of 100 µL per well. Before incubating the cells with resazurin according to the AlamarBlue assay, bright-field images were obtained with a widefield microscope (DMI 6000 SD, Leica Microsystems). The data point 5 nM of drug concentration represents the bottom plateau of the dose response curve for **DD1**-MMAE, **DFc**-MMAE and free MMAE on A431 cells, and for free MMAE on MCF7 cells (Fig. 3B-C). Within these samples, a few cells tolerate high drug dose in vitro, which are decisive for the minimal metabolic activity during the AlamarBlue assay. Adherent-looking cells were assumed to feature high drug tolerability (black arrows).

7 Animal data

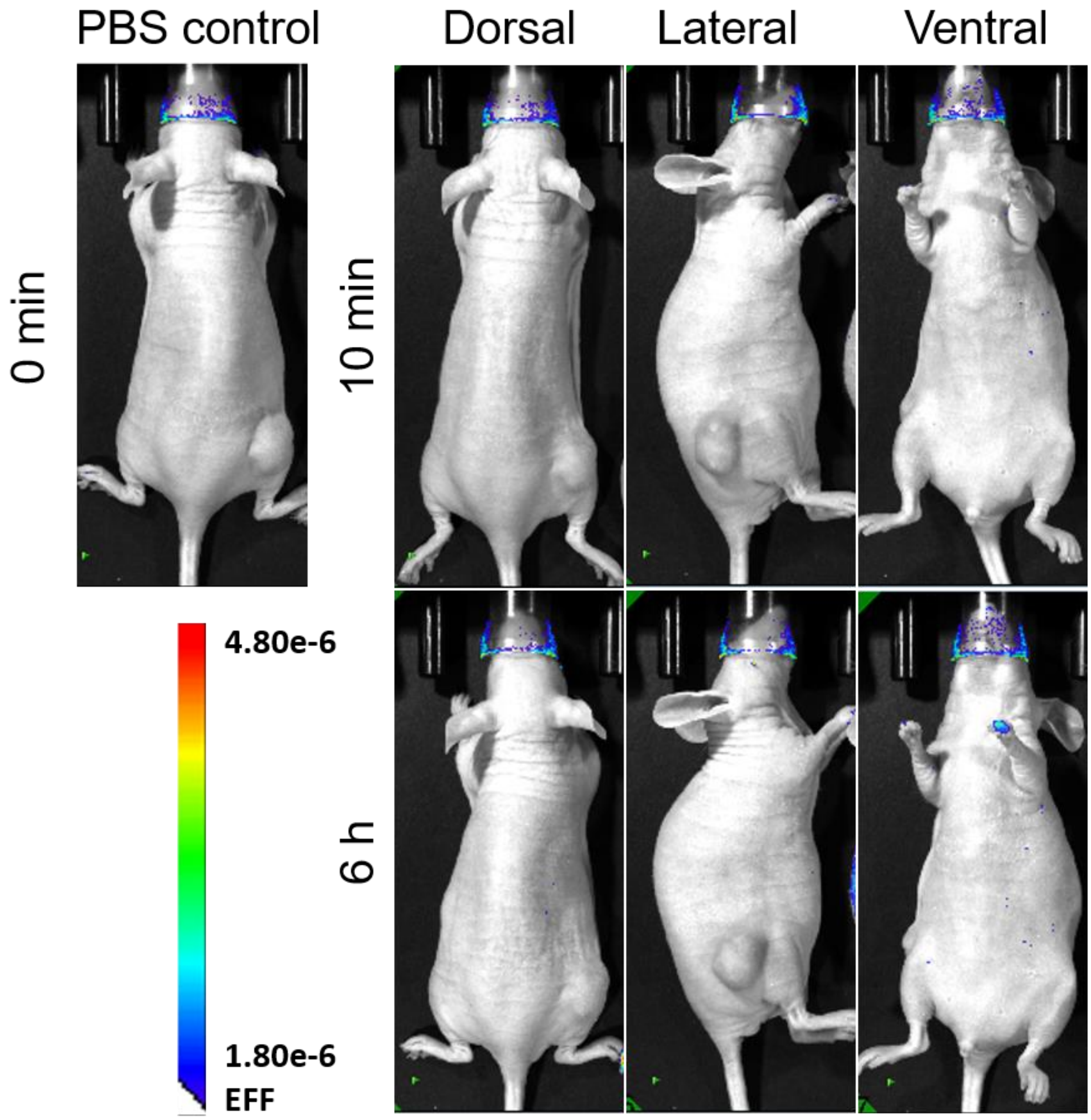

Figure S25: A431-xenografted mice were intravenously injected with a single dose of PBS. Whole-body imag-ing was performed at indicated time points post-injection using the Lago imager. Mouse was euthanized after 6 hours to proceed with *ex vivo* imaging.

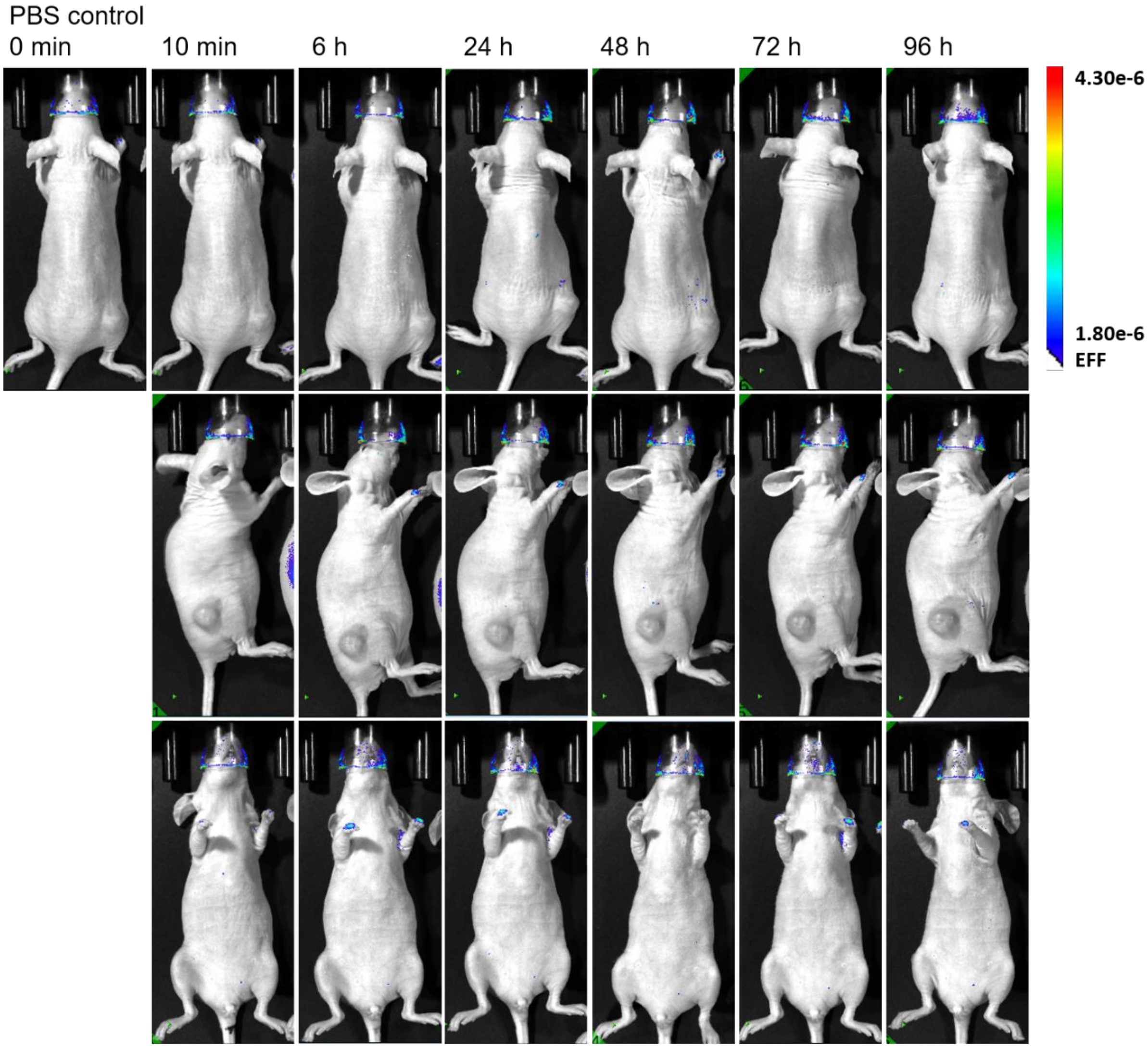

Figure S26: A431-xenografted mice were intravenously injected with a single dose of PBS. Whole-body imag-ing was performed at indicated time points post-injection using the Lago imager. Mouse was euthanized after 96 hours to proceed with *ex vivo* imaging.

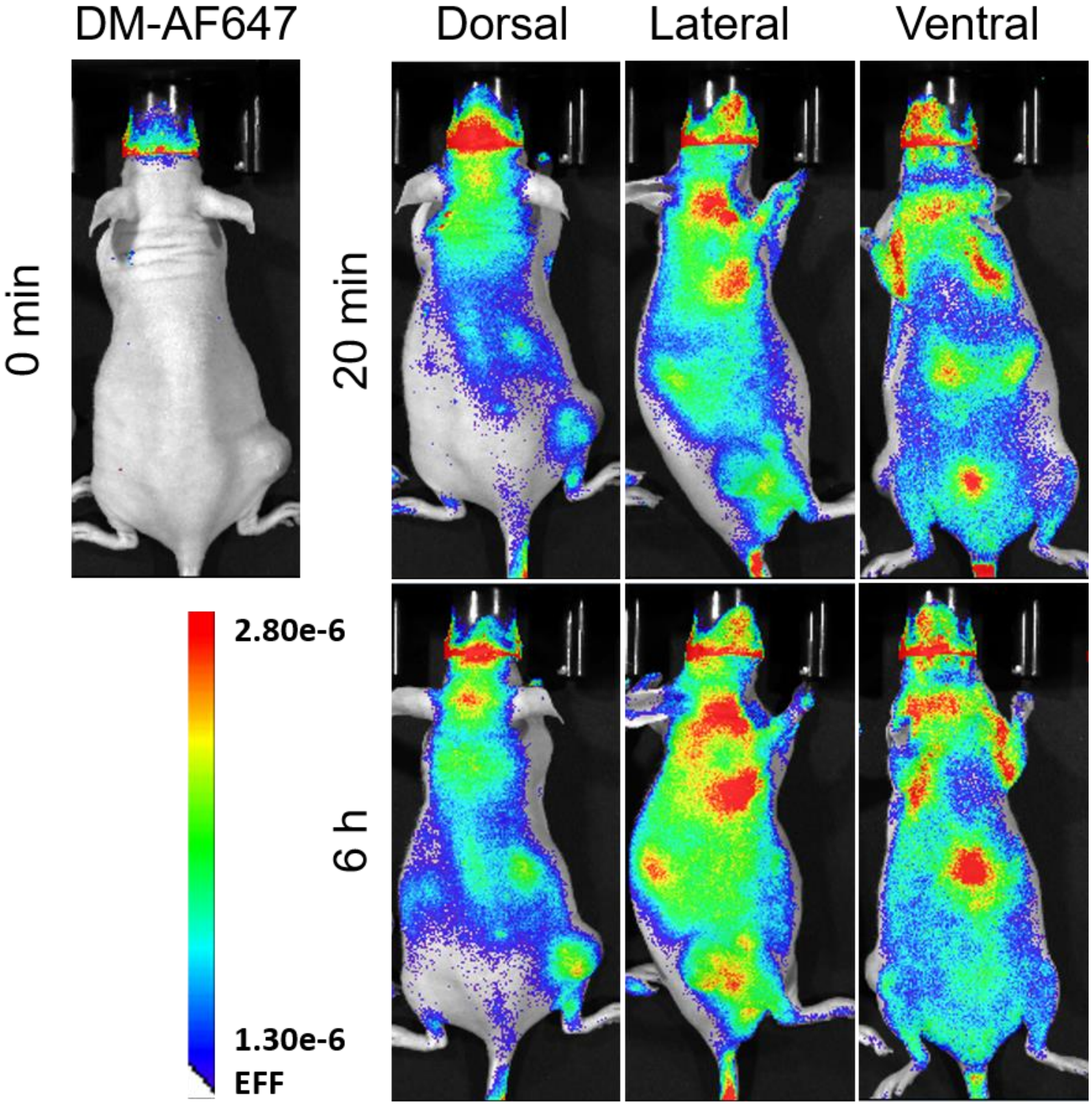

Figure S27: A431-xenografted mice were intravenously injected with a single dose of **DM**-Alexa Fluor 647. Whole-body imaging was performed at indicated time points post-injection using the Lago imager. Mouse was euthanized after 6 hours to proceed with *ex vivo* imaging.

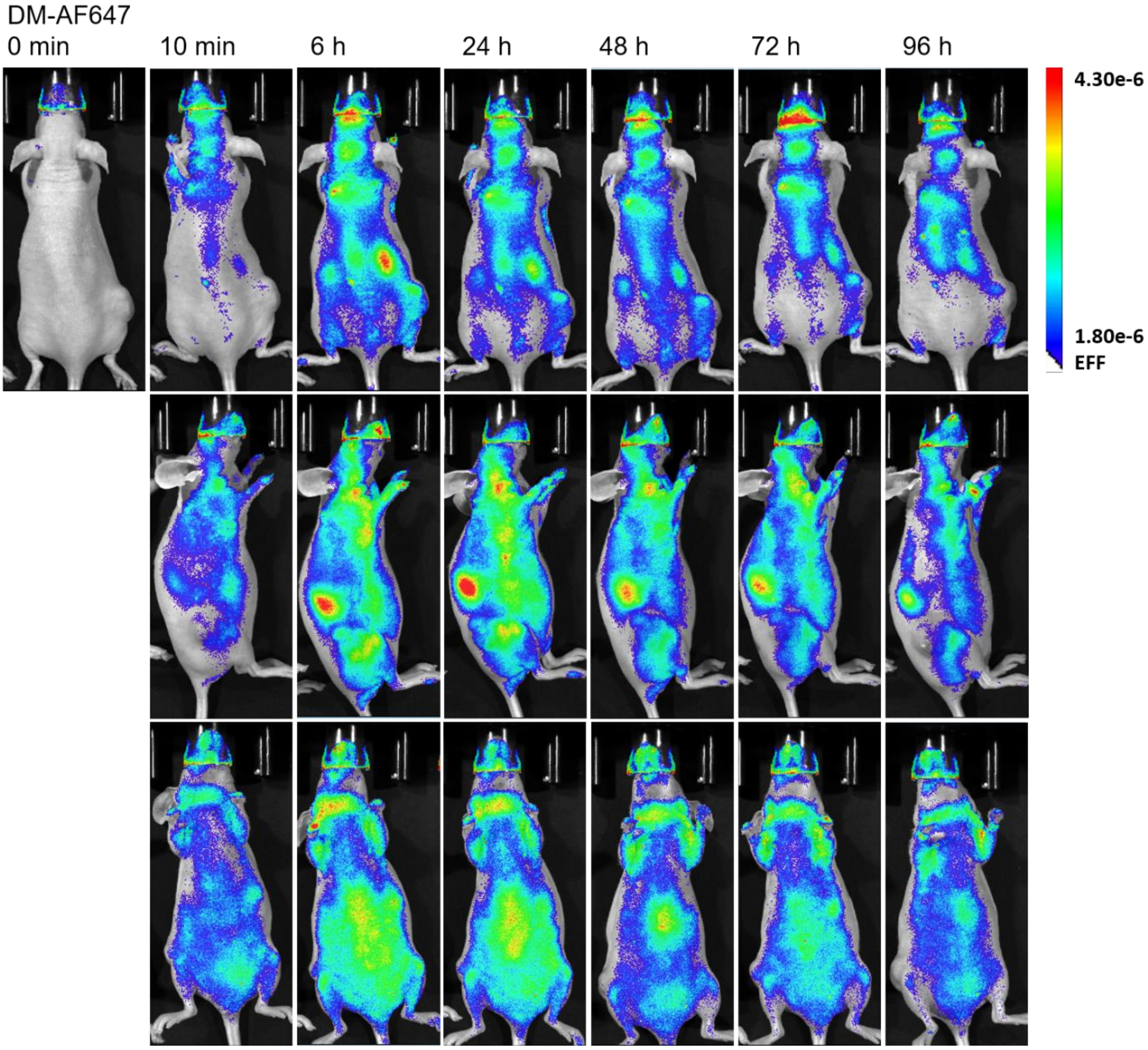

Figure S28: A431-xenografted mice were intravenously injected with a single dose of **DM**-Alexa Fluor 647. Whole-body imaging was performed at indicated time points post-injection using the Lago imager. Mouse was euthanized after 96 hours to proceed with *ex vivo* imaging.

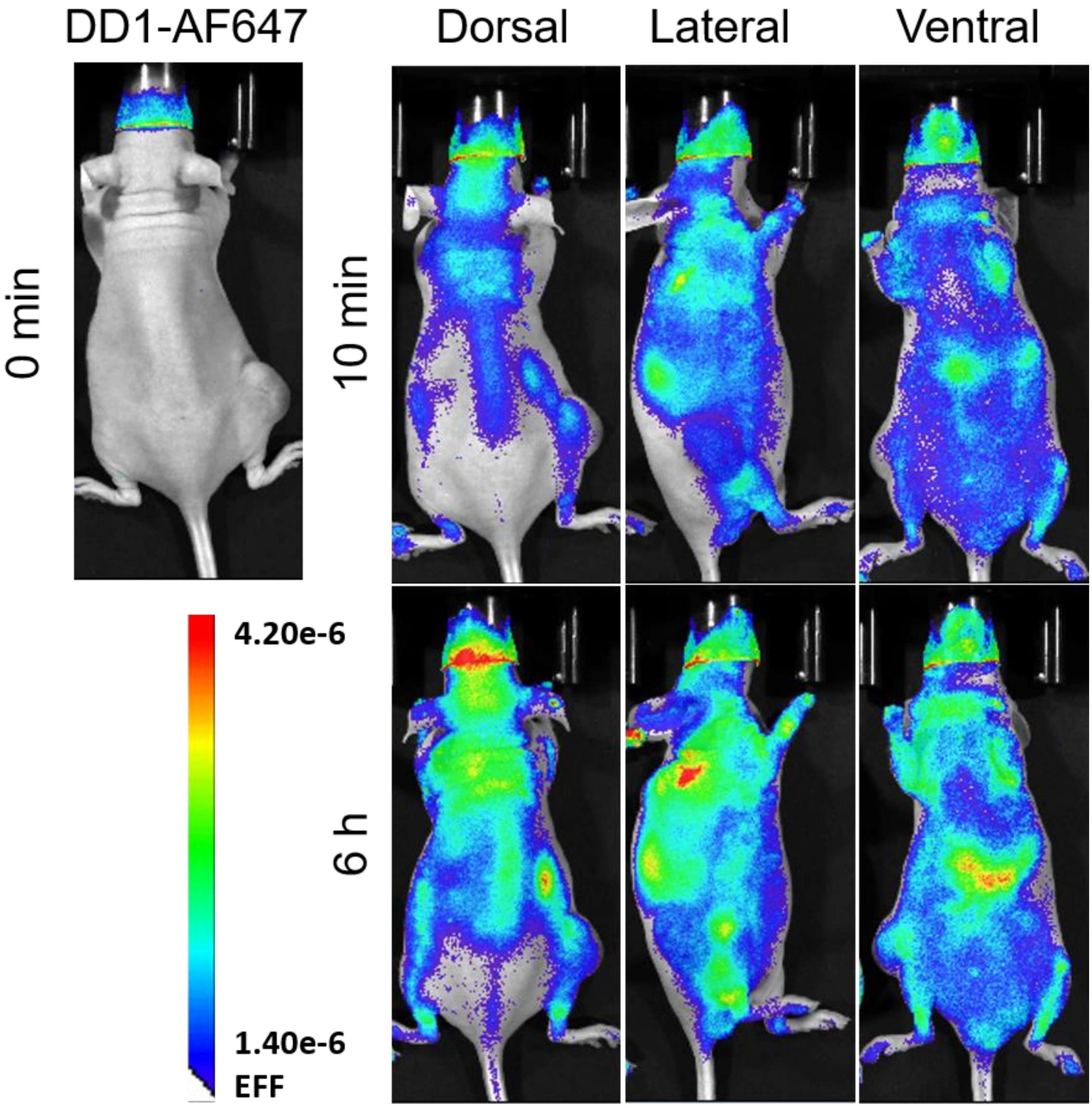

Figure S29: A431-xenografted mice were intravenously injected with a single dose of **DD1**-Alexa Fluor 647. Whole-body imaging was performed at indicated time points post-injection using the Lago imager. Mouse was euthanized after 6 hours to proceed with *ex vivo* imaging.

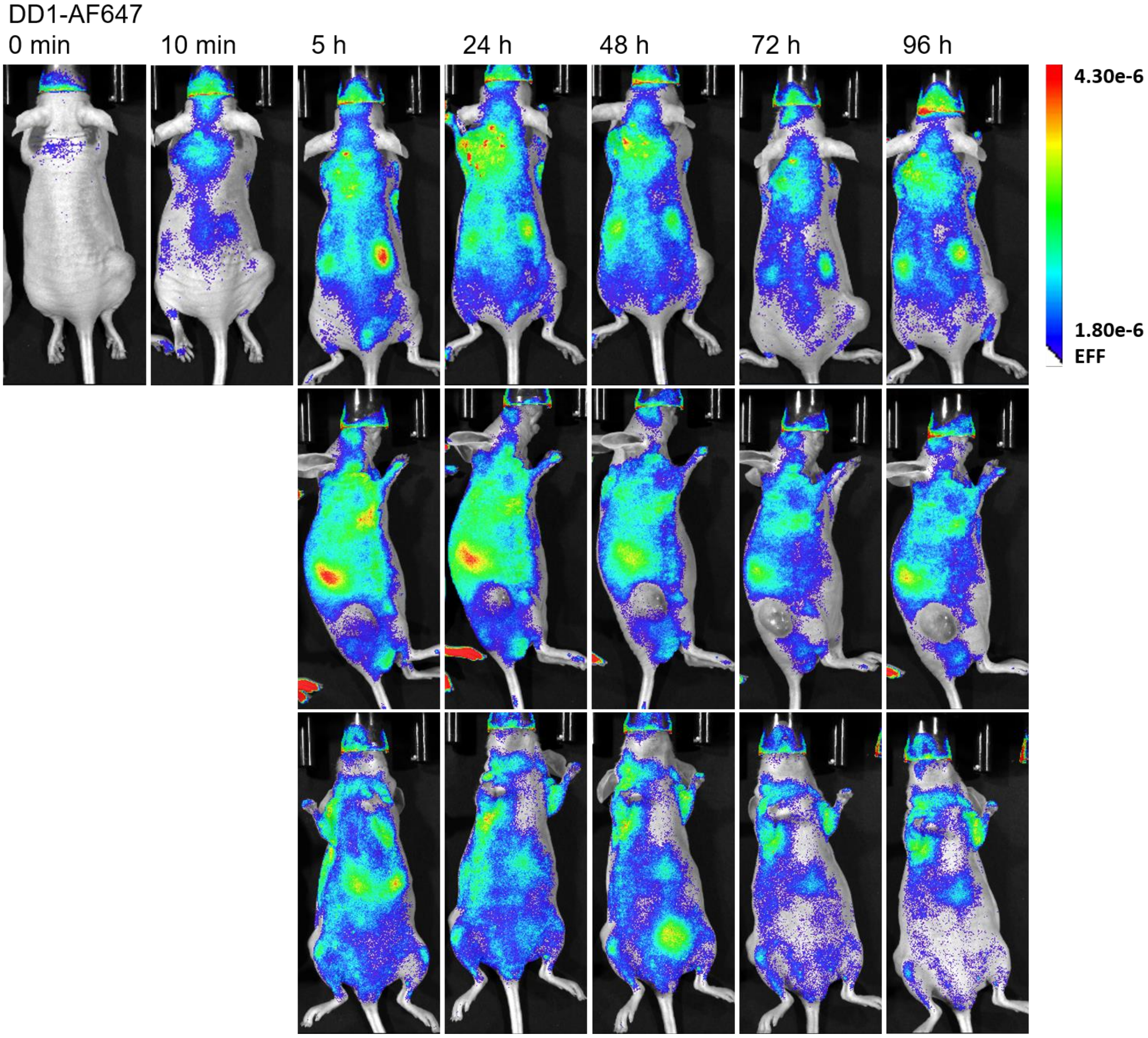

Figure S30: A431-xenografted mice were intravenously injected with a single dose of **DD1**-Alexa Fluor 647. Whole-body imaging was performed at indicated time points post-injection using the Lago imager. Mouse was euthanized after 96 hours to proceed with *ex vivo* imaging.

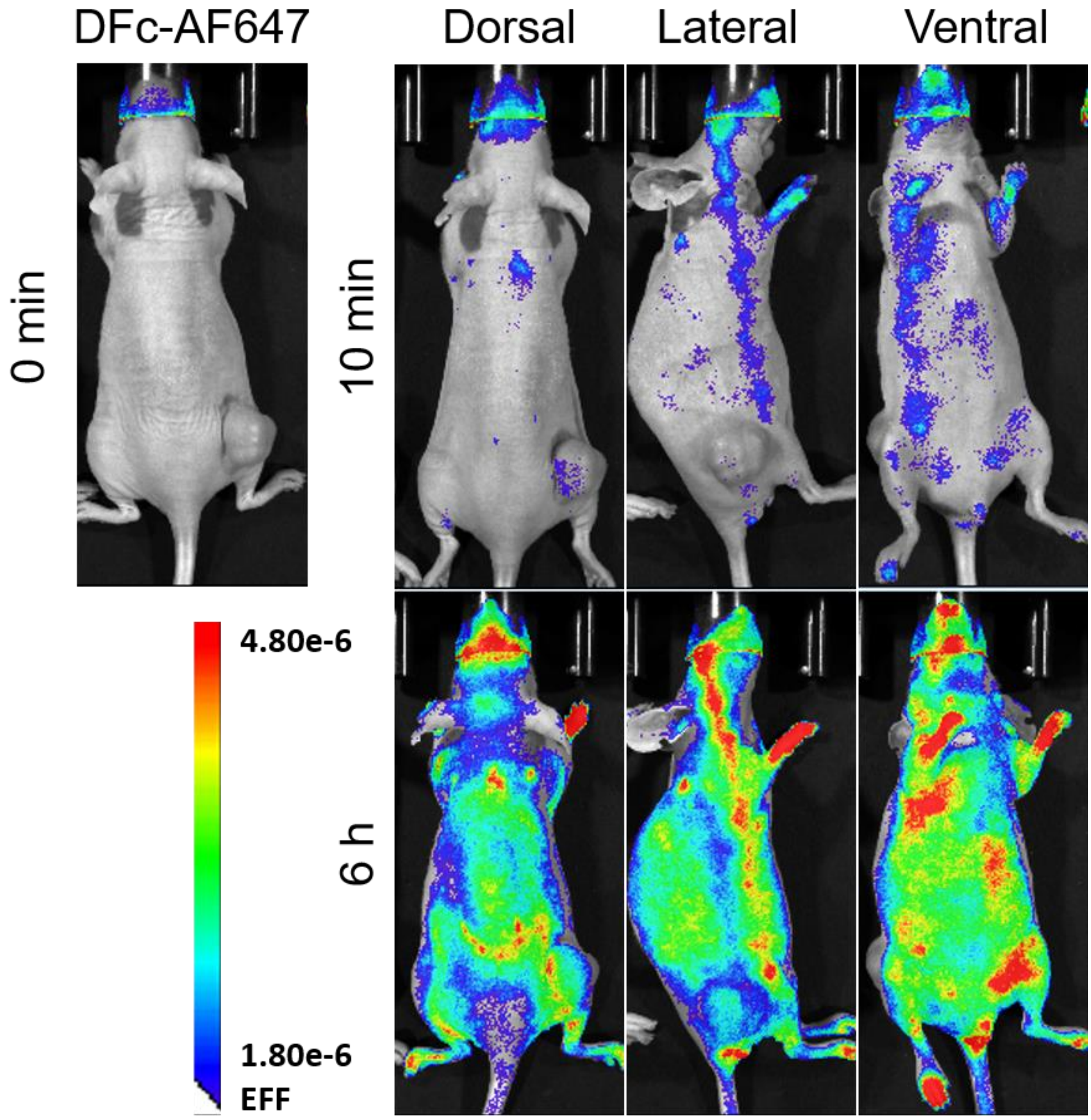

Figure S31: A431-xenografted mice were intravenously injected with a single dose of **DFc**-Alexa Fluor 647. Whole-body imaging was performed at indicated time points post-injection using the Lago imager. Mouse was euthanized after 6 hours to proceed with *ex vivo* imaging.

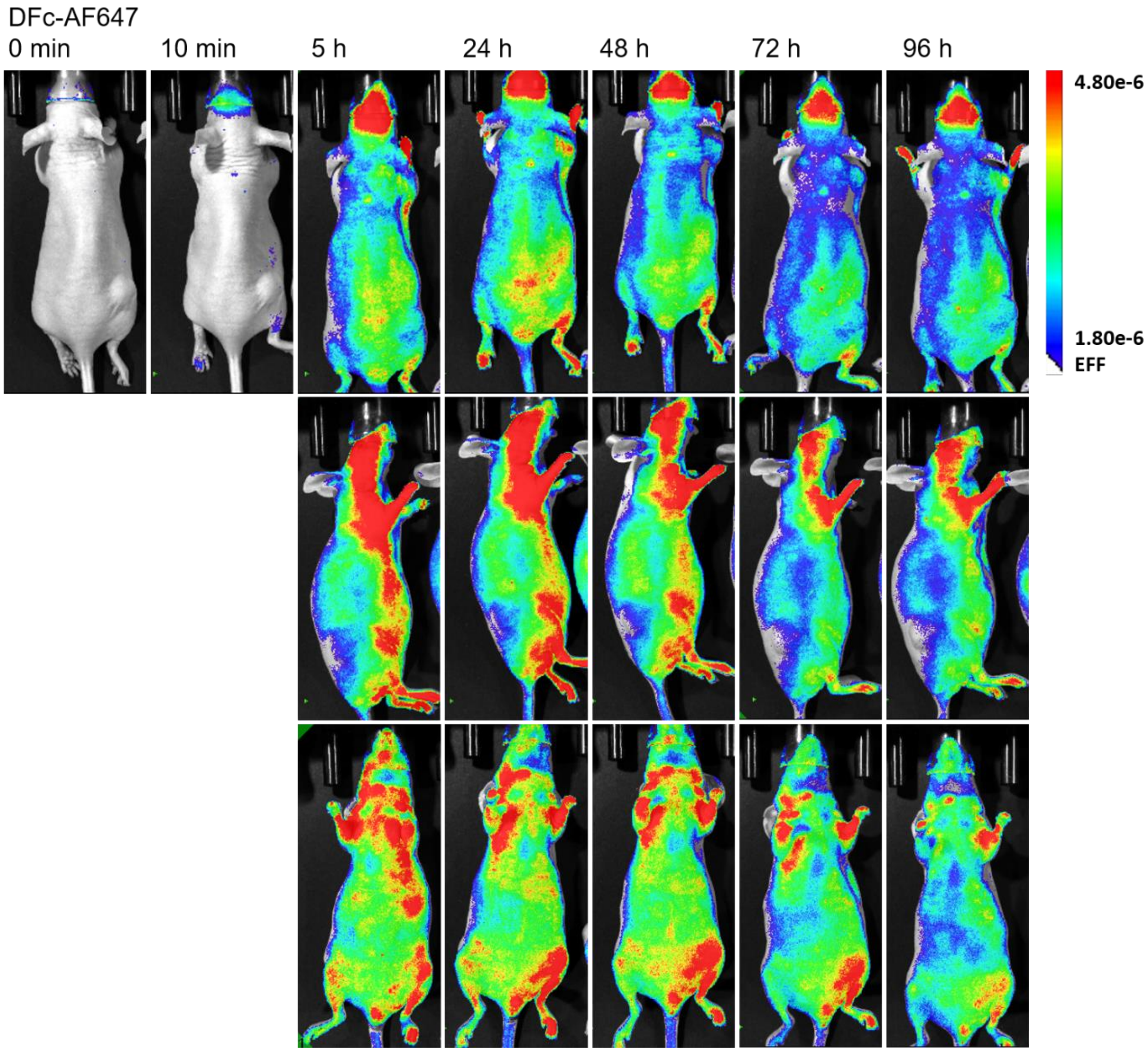

Figure S32: A431-xenografted mice were intravenously injected with a single dose of **DFc**-Alexa Fluor 647. Whole-body imaging was performed at indicated time points post-injection using the Lago imager. Mouse was euthanized after 96 hours to proceed with *ex vivo* imaging.

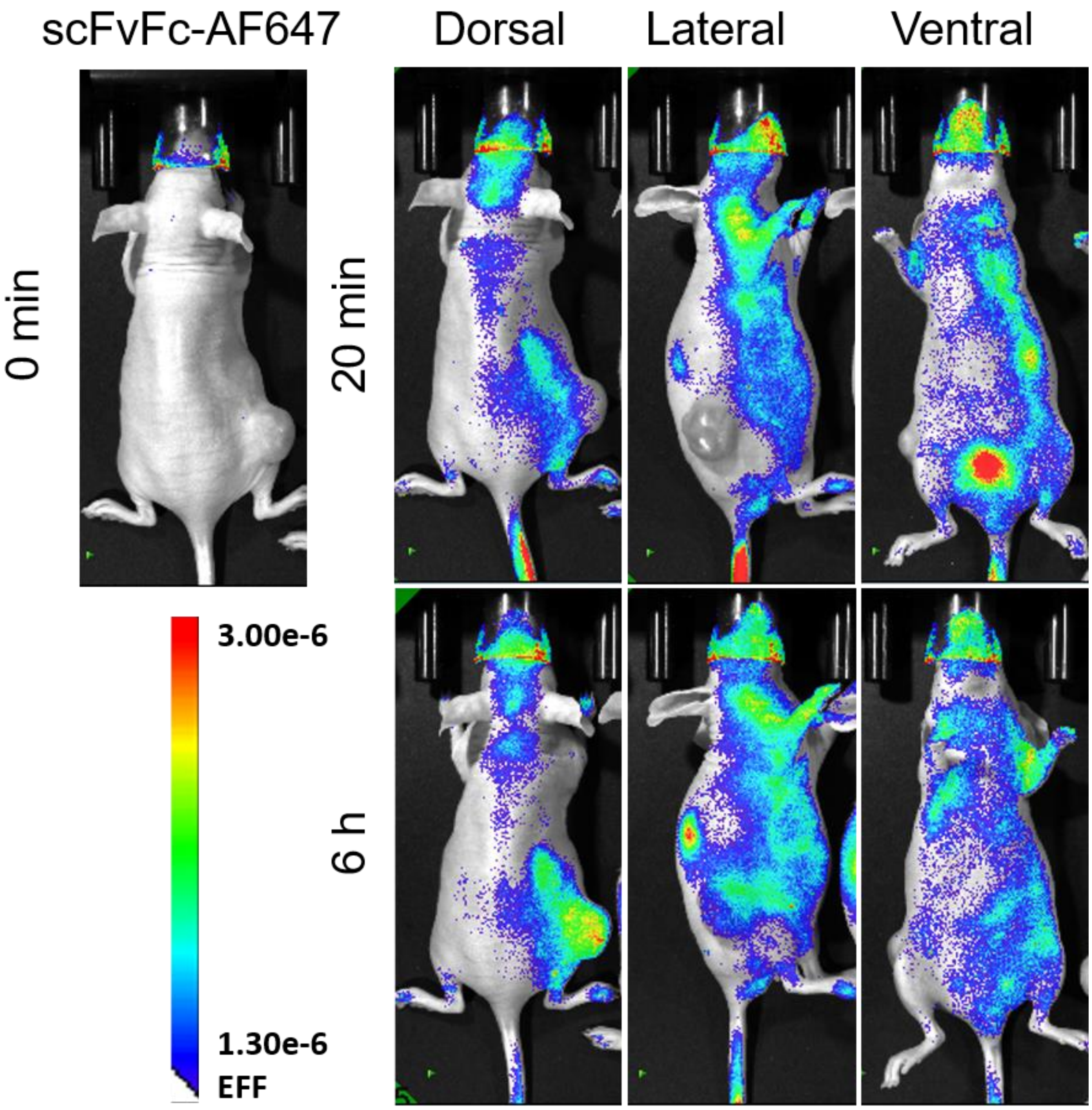

Figure S33: A431-xenografted mice were intravenously injected with a single dose of **scFvFc**-Alexa Fluor 647. Whole-body imaging was performed at indicated time points post-injection using the Lago imager. Mouse was euthanized after 6 hours to proceed with *ex vivo* imaging.

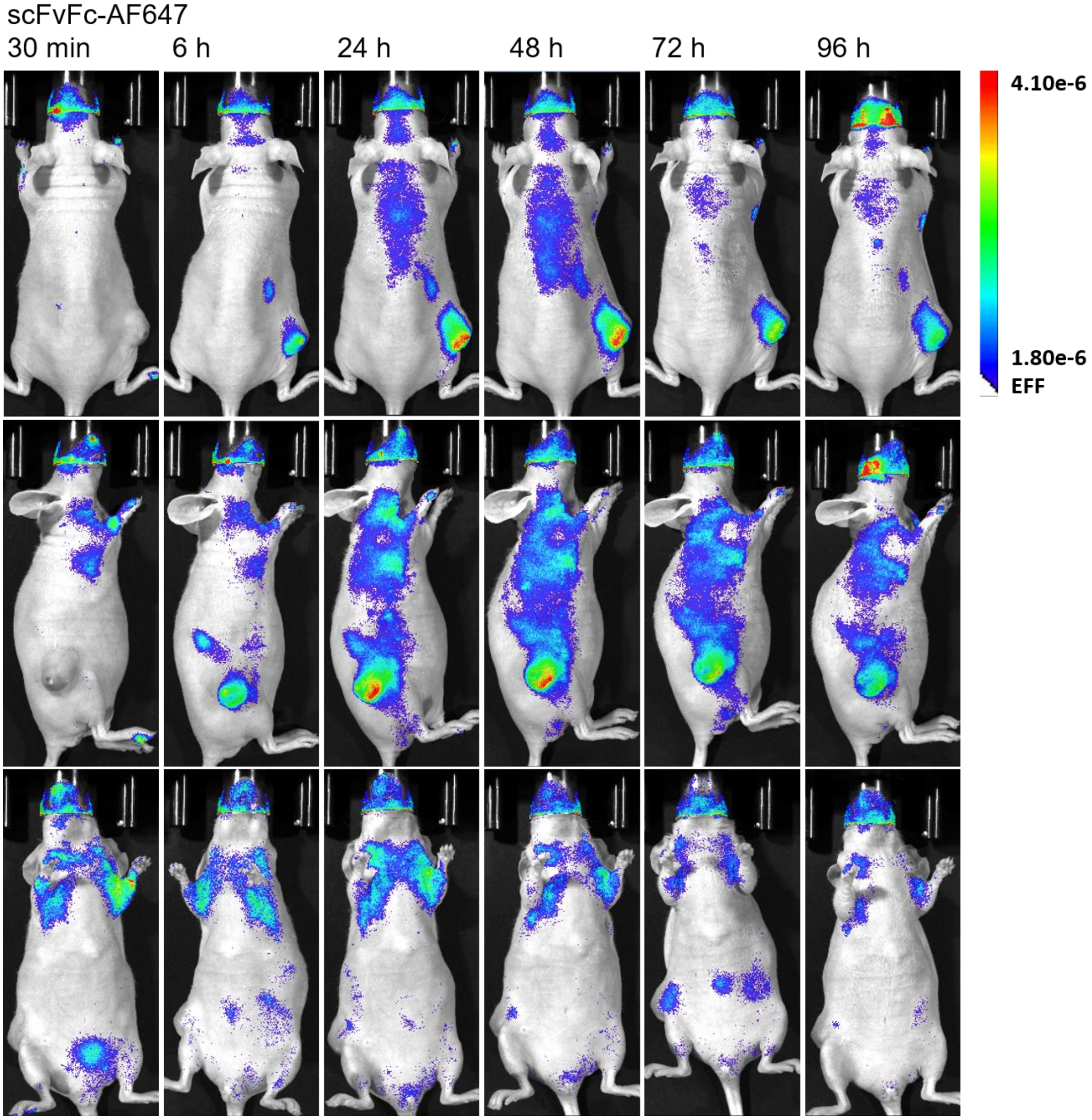

Figure S34: A431-xenografted mice were intravenously injected with a single dose of **scFvFc**-Alexa Fluor 647. Whole-body imaging was performed at indicated time points post-injection using the Lago imager. Mouse was euthanized after 96 hours to proceed with *ex vivo* imaging.

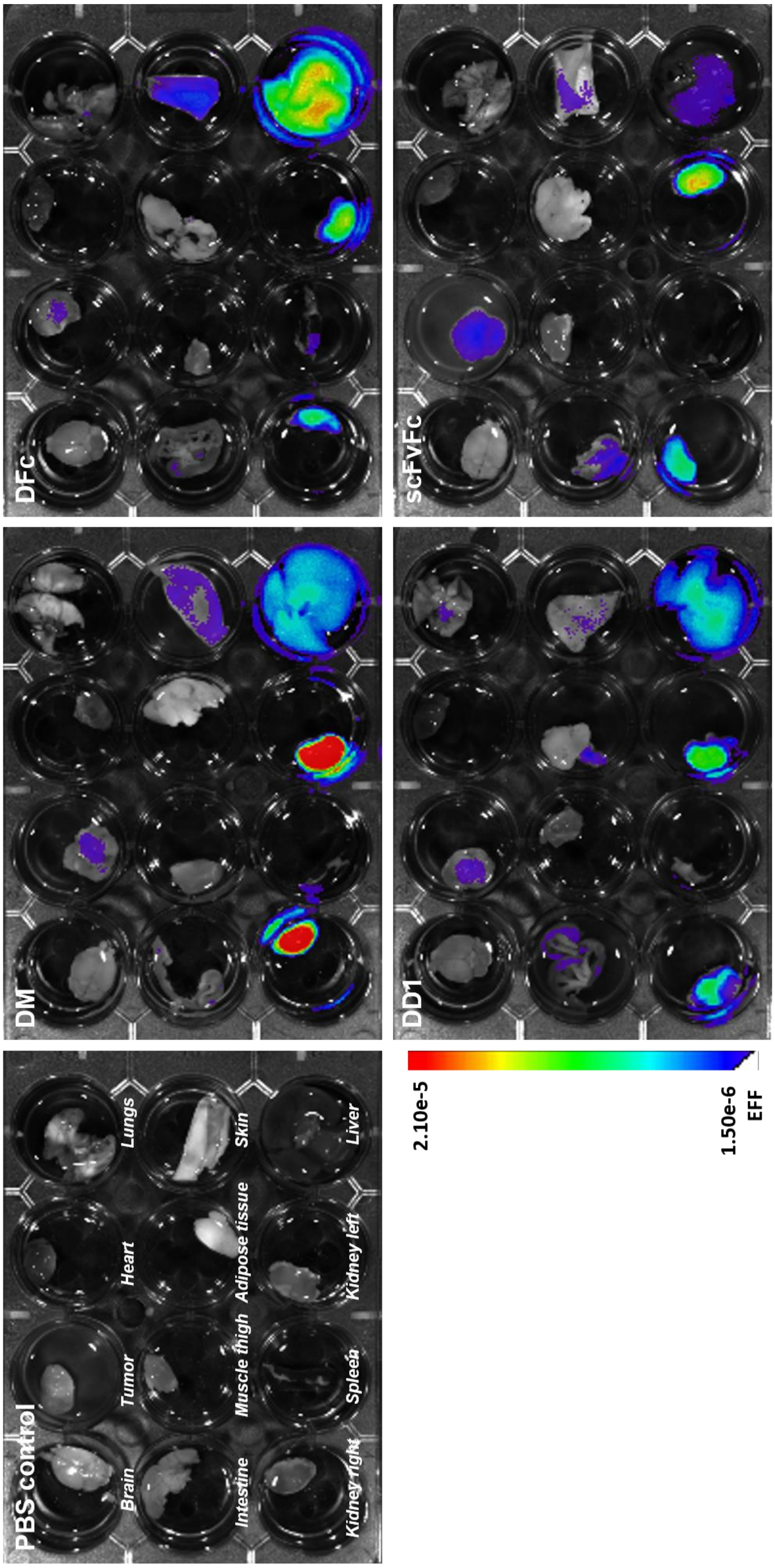

Figure S35: *Ex vivo* imaging of anti-EGFR dye-conjugates. A431-xenografted mice were intravenously injected with Alexa Fluor 647-labeled **DM**, **DD1**, **DFc**, **scFvFc** dye-conjugates, or PBS solution. Organs were collected from PFA-perfused mice 96 hours post-injection.

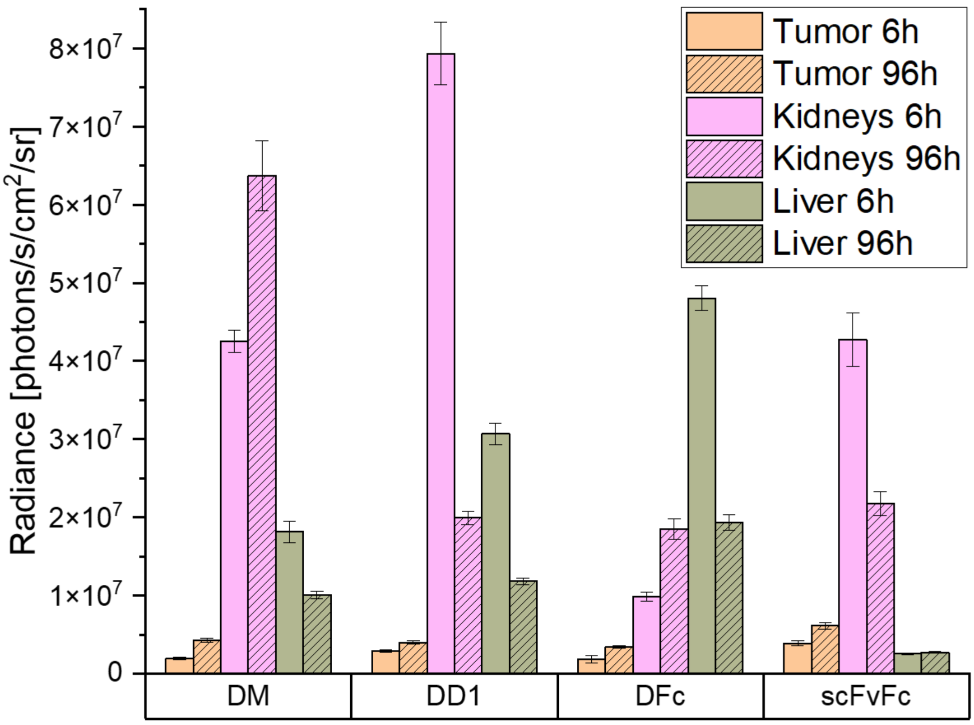

Figure S36: The mean signal intensity of Alexa Fluor 647 for tumor, liver and kidneys was quantified from *ex vivo* images after 6 and 96 hours using Aura imaging software. Autofluorescence subtraction was separately done for each organ (mean value of both kidneys was considered). Error bars represent standard error of the mean.

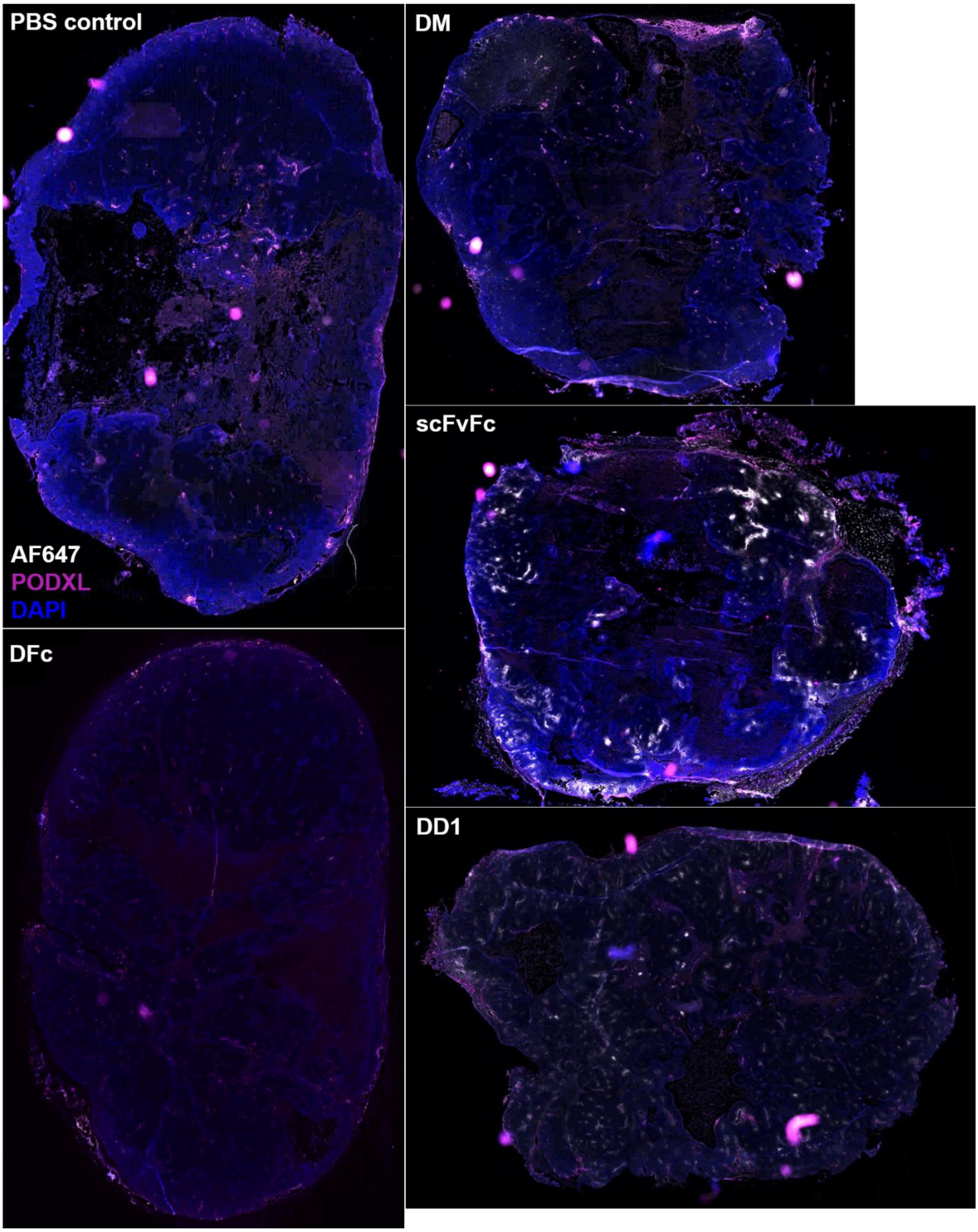

Figure S37: A431-xenografted mice were intravenously injected with Alexa Fluor 647-labeled **DM**, **DD1**, **DFc**, **scFvFc** dye-conjugates, or PBS solution. Tumors were collected from PFA-perfused mice 6 hours post-injec-tion. Immunofluorescence of complete tumor slide scans of each treatment group shows Alexa Fluor 647 (white), podocalyxin (magenta) and DAPI (blue) staining.

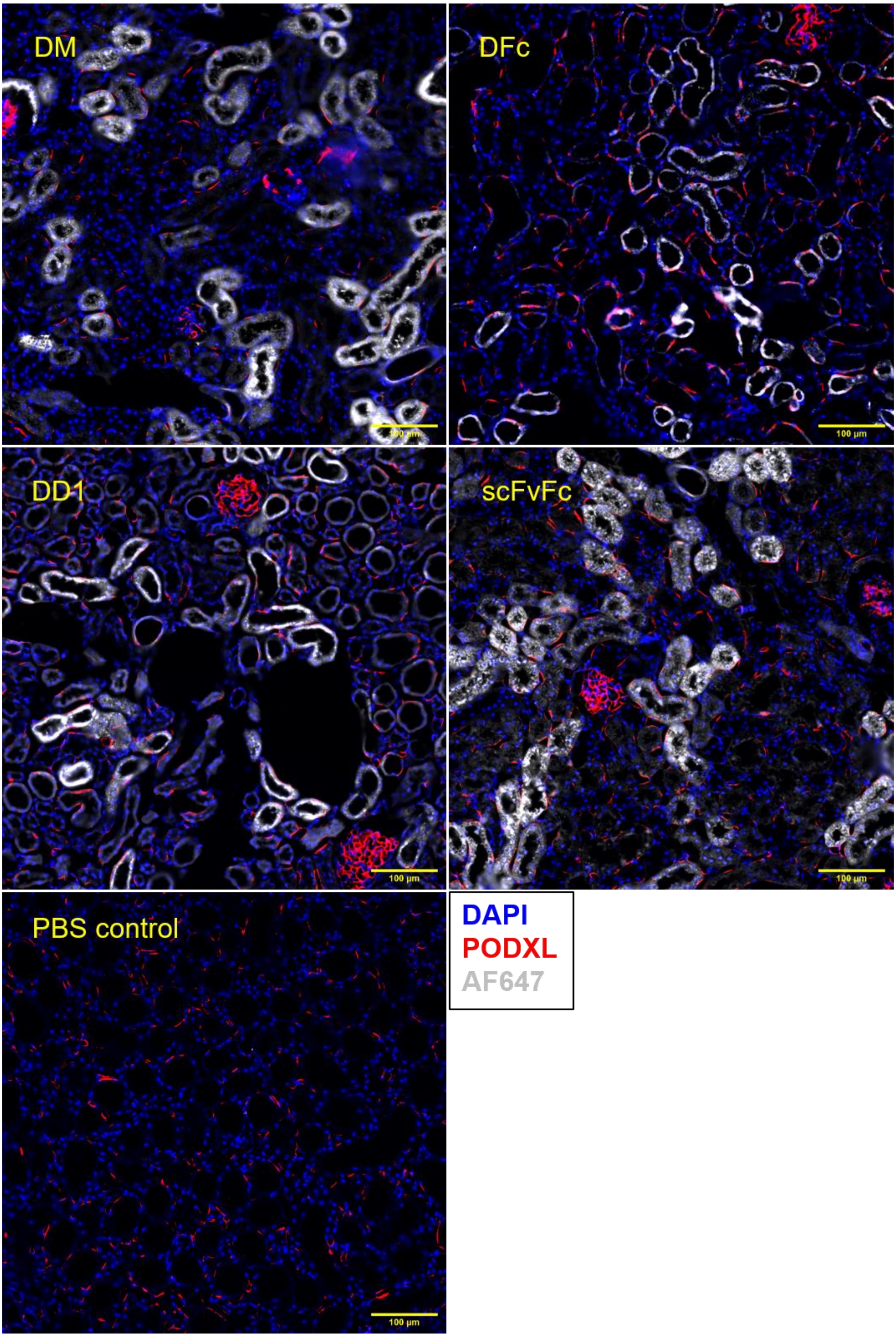

Figure S38: A431-xenografted mice were intravenously injected with Alexa Fluor 647-labeled **DM**, **DD1**, **DFc**, **scFvFc** dye-conjugates, or PBS solution. Kidneys were collected from PFA-perfused mice 6 hours post-injec-tion. Immunofluorescence of representative kidney section of each treatment group shows Alexa Fluor 647 (white), podocalyxin (magenta) and DAPI (blue) staining.

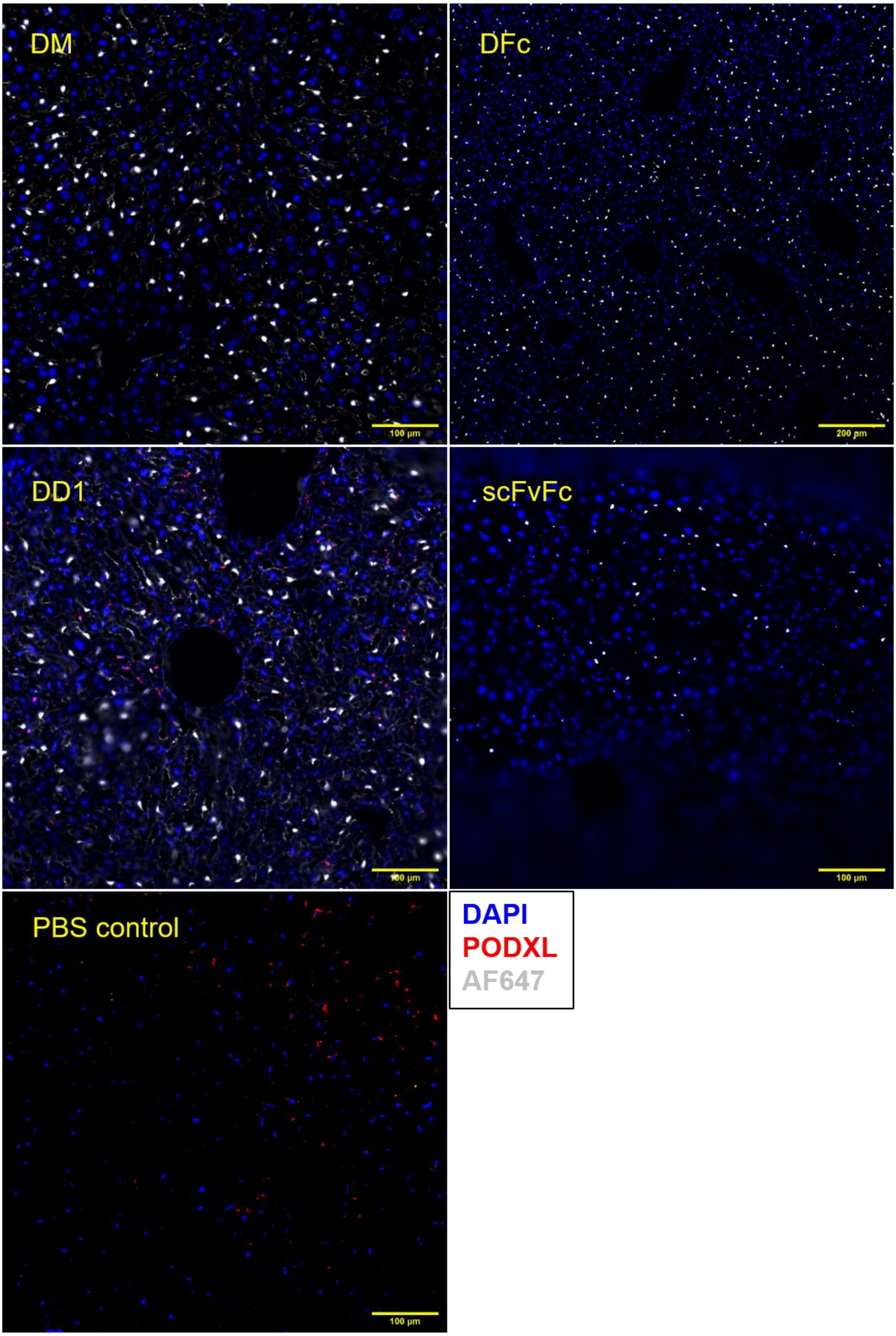

Figure S39: A431-xenografted mice were intravenously injected with Alexa Fluor 647-labeled **DM**, **DD1**, **DFc**, **scFvFc** dye-conjugates, or PBS solution. Livers were collected from PFA-perfused mice 6 hours post-injec-tion. Immunofluorescence of representative liver section of each treatment group shows Alexa Fluor 647 (white), podocalyxin (magenta) and DAPI (blue) staining.

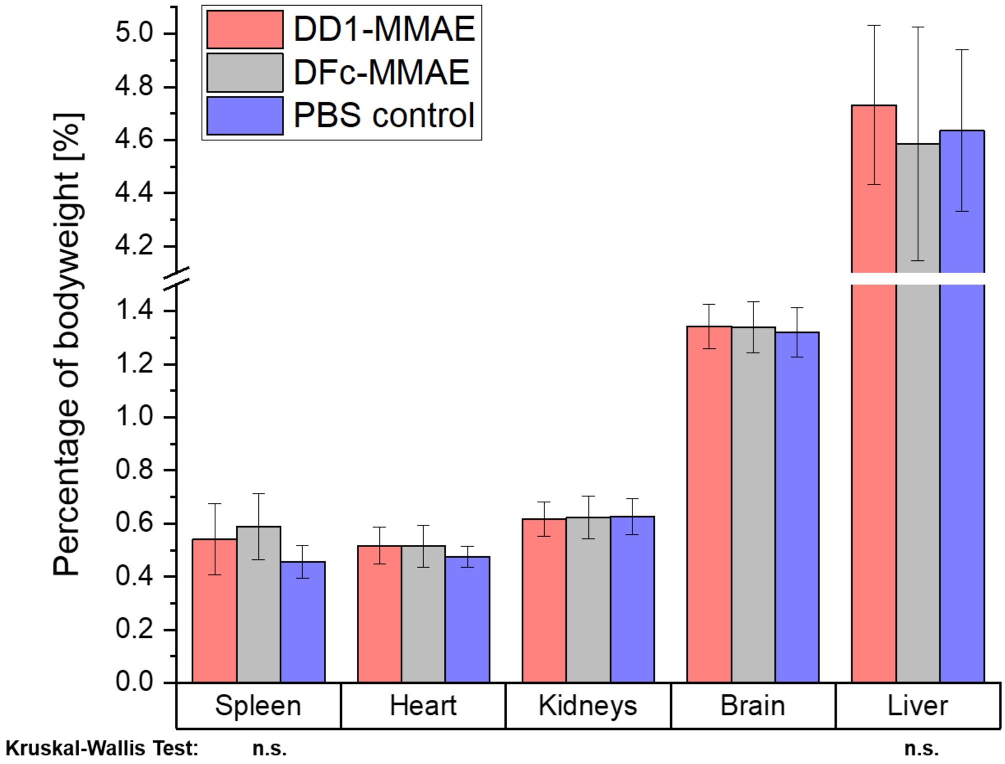

Figure S40: Nude mice bearing A431 tumors were intravenously injected twice weekly with 100 µL of drug-conjugate **DD1**-MMAE (6.5 mg/kg in PBS), **DFc**-MMAE (6.5 mg/kg in PBS), or PBS only with four injections in total. The treatment started at a tumor volume of 90 mm³. Animals were euthanized, organs were dis-sected and weighted before freezing. Organ weight was normalized to the body weight. Error bars indicate the SD; n = 5 mice per group (mean value of both kidneys was considered). Kruskal-Wallis rank sum test with a $P < 0.05$ level of significance was used to estimate statistical significance between categories.

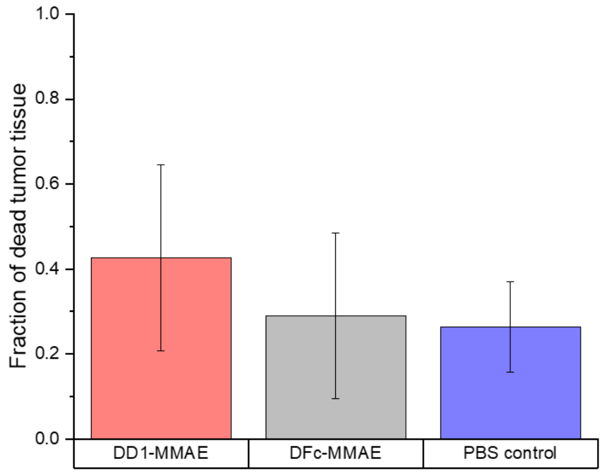

Figure S41: Nude mice bearing A431 tumors were intravenously injected twice weekly with 100 µL of drug-conjugate **DD1**-MMAE (6.5 mg/kg in PBS), **DFc**-MMAE (6.5 mg/kg in PBS), or PBS only with four injections in total. The treatment started at a tumor volume of 90 mm³. Animals were euthanized, tumors were dis-sected, snap-frozen and tissue sections were stained for EGFR and DAPI by immunofluorescence to deter-mine the necrotic area. Necrotic areas were normalized to the total area of the tumor section.

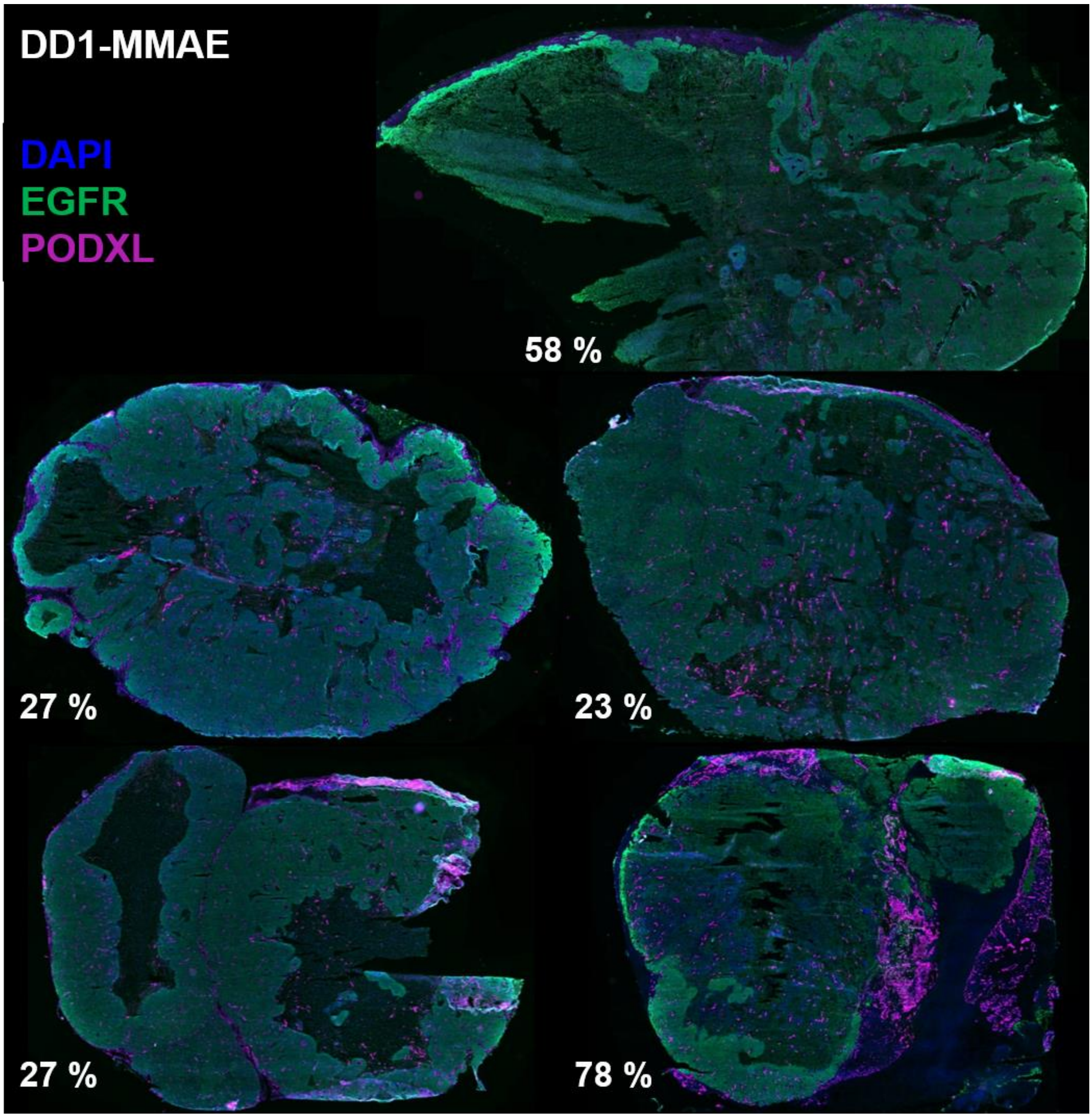

Figure S42: Immunofluorescence of complete tumor slide scans of **DD1**-MMAE treatment group shows EGFR (green), podocalyxin (magenta) and DAPI (blue). Necrotic areas were normalized to the total area of the tumor section. Percentages of necrotic areas are given. n = 5 tumors.

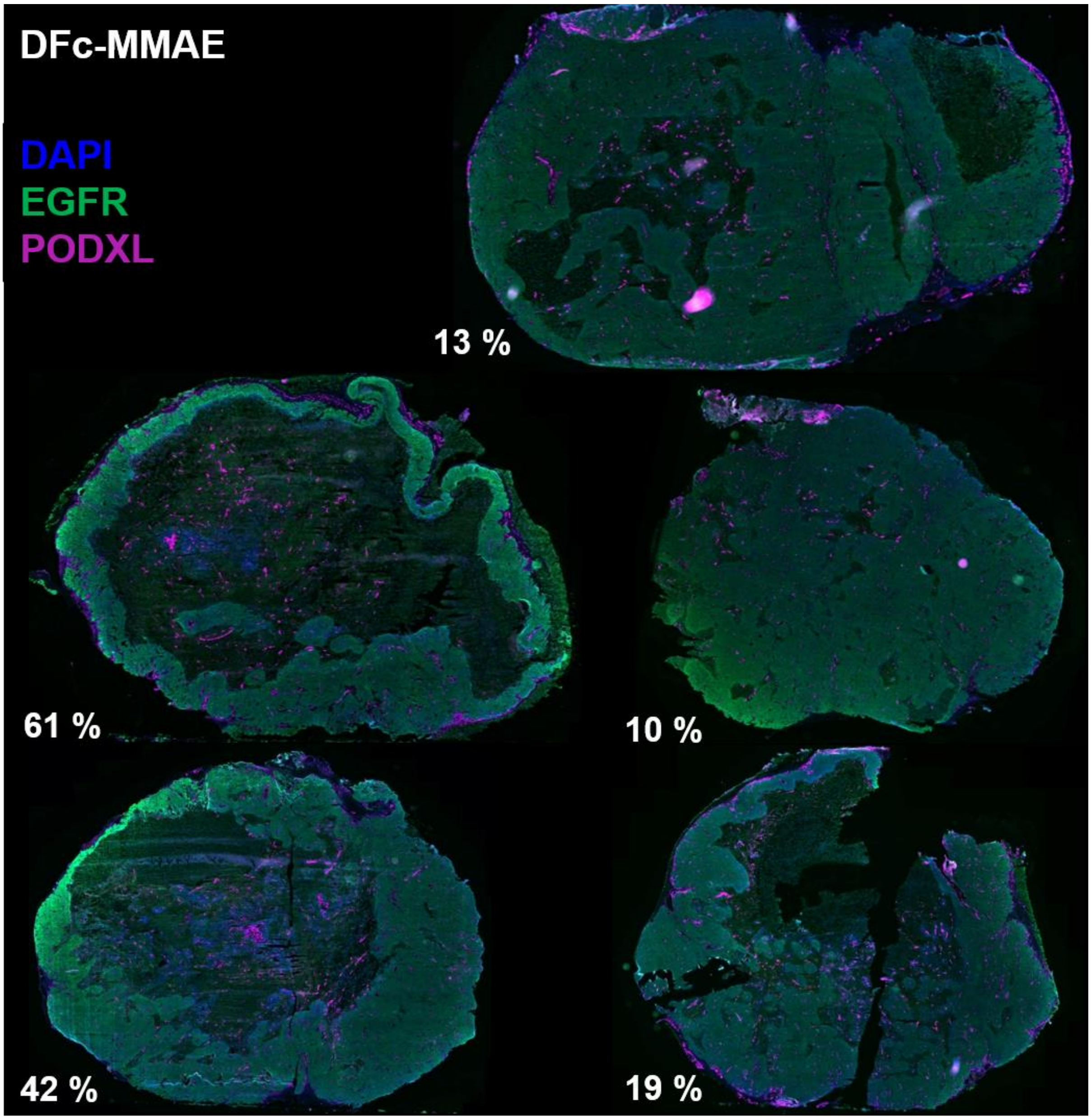

Figure S43: Immunofluorescence of complete tumor slide scans of **DFc**-MMAE treatment group shows EGFR (green), podocalyxin (magenta) and DAPI (blue). Necrotic areas were normalized to the total area of the tumor section. Percentages of necrotic areas are given. n = 5 tumors.

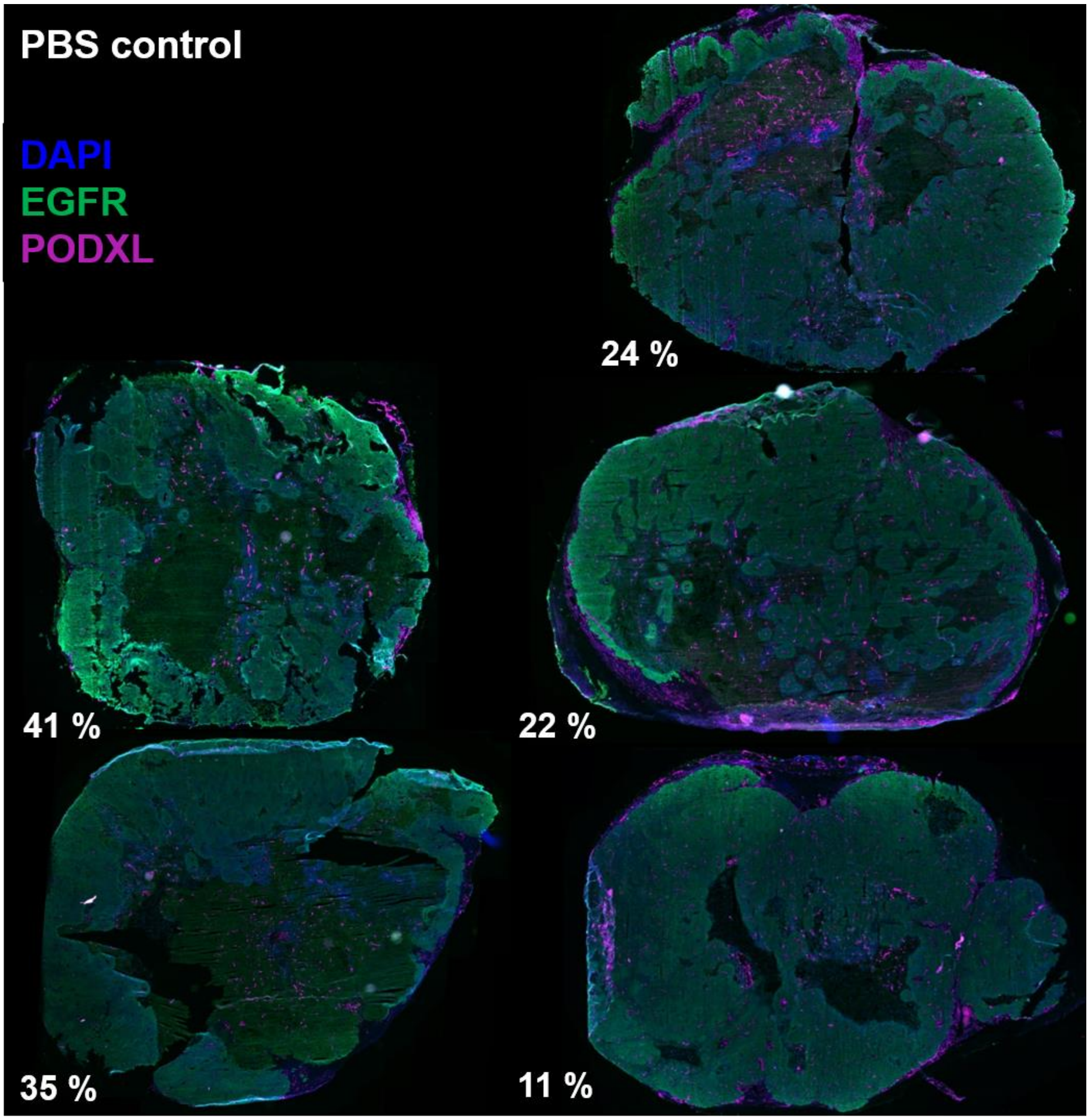

Figure S44: Immunofluorescence of complete tumor slide scans of PBS control group shows EGFR (green), podocalyxin (magenta) and DAPI (blue). Necrotic areas were normalized to the total area of the tumor sec-tion. Percentages of necrotic areas are given. n = 5 tumors.

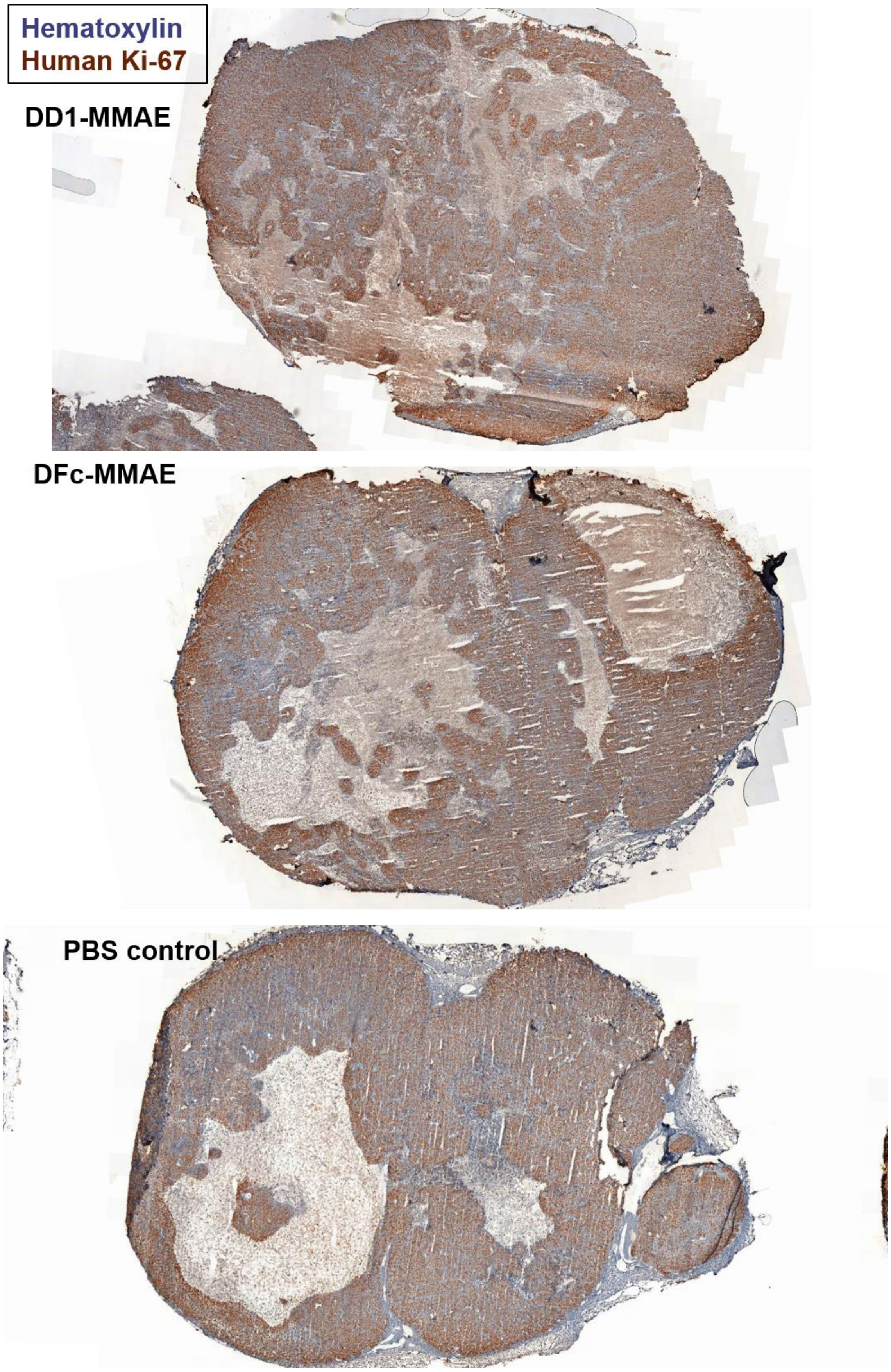

Figure S45: Immunohistochemistry of complete tumor slide scans of **DD1**-MMAE, **DFc**-MMAE, PBS treatment groups shows human Ki-67 (brown) and was counter stained with hematoxylin (blue). One representative slide scan is presented from each group.

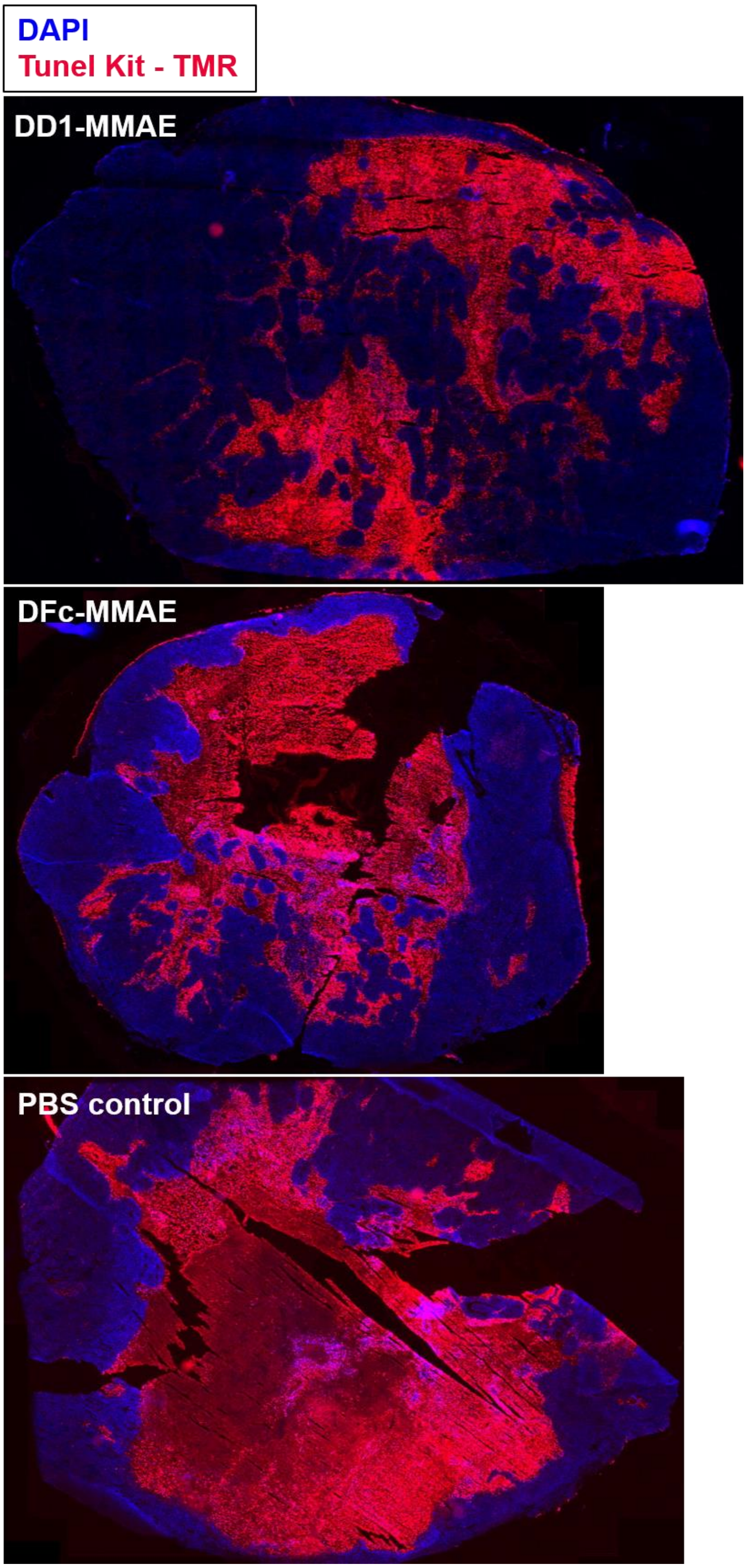

Figure S46: TUNEL (terminal deoxynucleotidyl transferase dUTP nick end labeling) assay (red) of complete tumor slide scans of **DD1**-MMAE, **DFc**-MMAE, PBS treatment groups Counter staining was carried out using DAPI. One representative slide scan is presented from each group.

8 LC-MS data

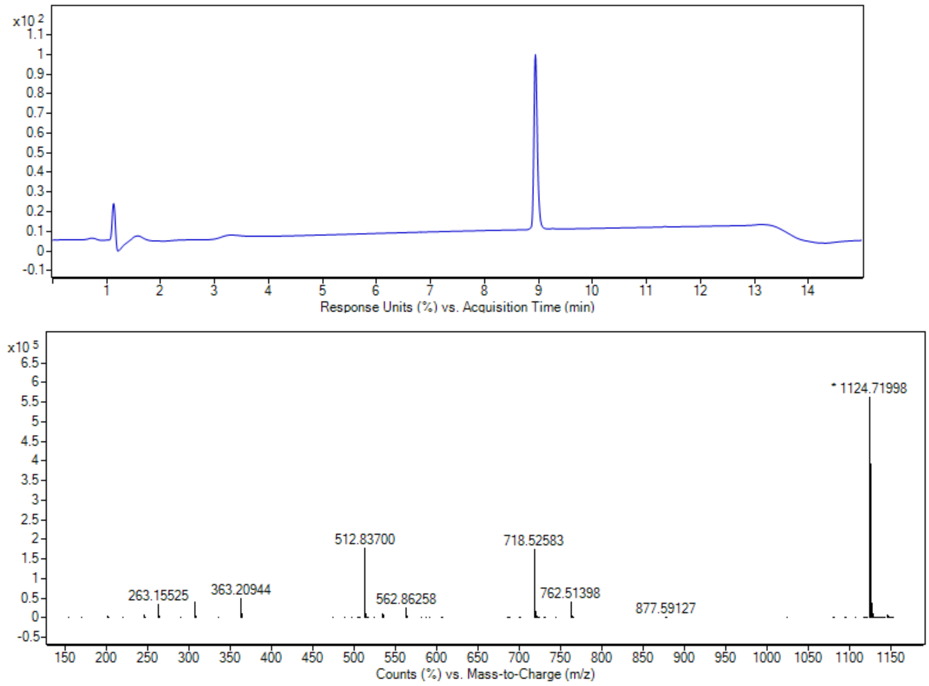

C18 RP-HPLC-MS analysis of Boc-Cit-PAB-MMAE recorded at 220 nm. Top: Elution diagram with a linear acetonitrile gradient (5-95% + 0.1% formic acid). Bottom: Associated ESI-MS spectrum of the elution band.

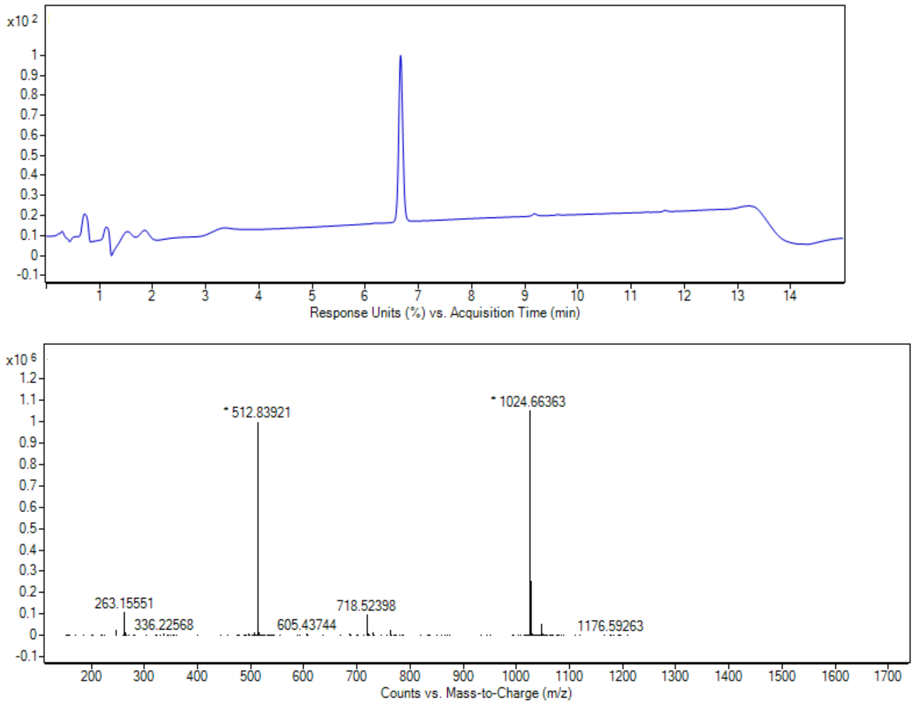

C18 RP-HPLC-MS analysis of H-Cit-PAB-MMAE recorded at 220 nm. Top: Elution diagram with a linear acetonitrile gradient (5-95% + 0.1% formic acid). Bottom: Associated ESI-MS spectrum of the elution band.

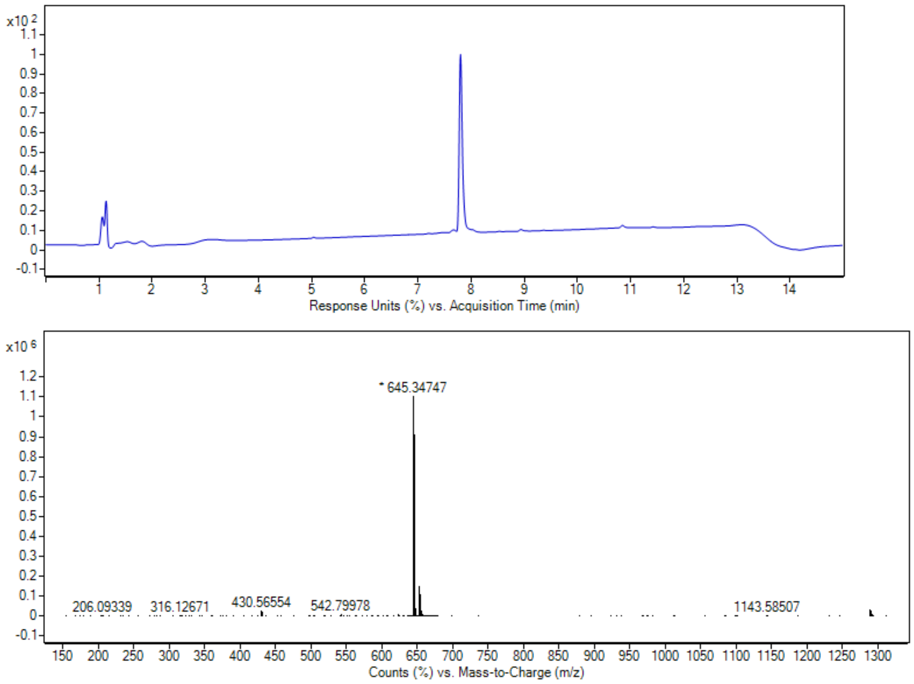

C18 RP-HPLC-MS analysis of DBCO-PEG$_2$-Lys(mPEG$_{10}$)-βAla-Val-OH recorded at 220 nm. Top: Elution diagram with a linear acetonitrile gradient (5-95% + 0.1% formic acid). Bottom: Associated ESI-MS spectrum of the elution band.

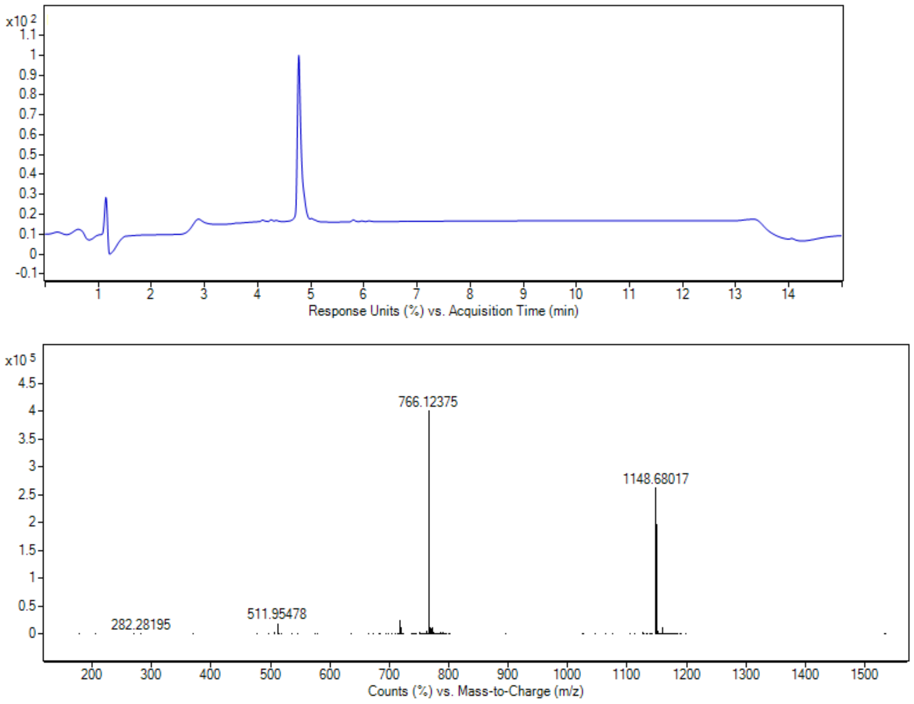

C18 RP-HPLC-MS analysis of DBCO-PEG$_2$-Lys(mPEG$_{10}$)-βAla-Val-Ala-PAB-MMAE recorded at 220 nm. Top: Elution diagram with an isocratic acetonitrile gradient (+ 0.1% formic acid). Bottom: Associated ESI-MS spectrum of the elution band.

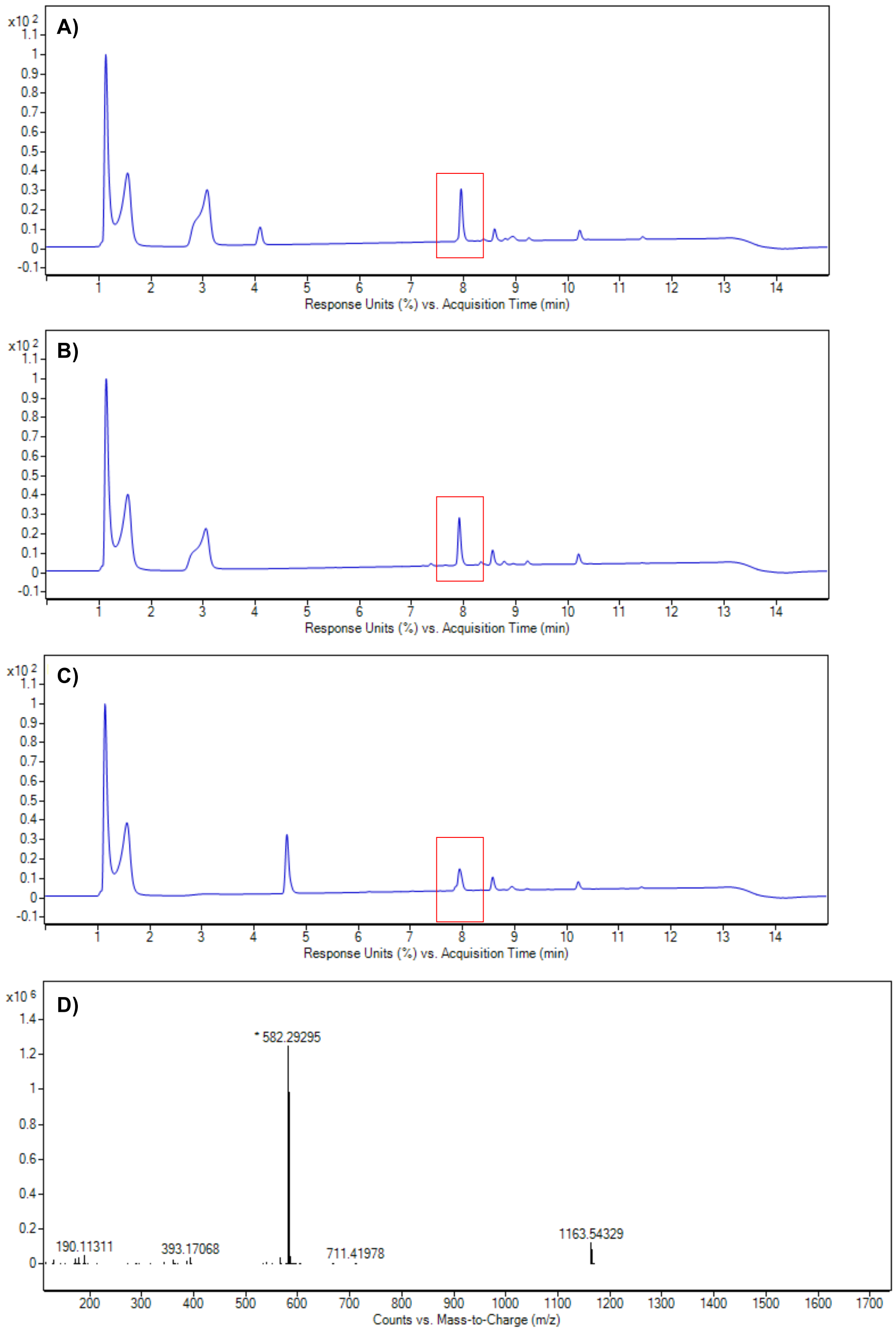

C18 RP-HPLC-MS analysis of the reaction mixture of Fmoc-βAla-Val-Cit-OMe recorded at 220 nm with a linear acetonitrile gradient (5-95% + 0.1% formic acid). A) Reaction with HATU as coupling reagent. B) Reaction with PyAOP as coupling reagent. C) Reaction with DEPBT as coupling reagent. D) Associated ESI-MS spectrum of the elution band.

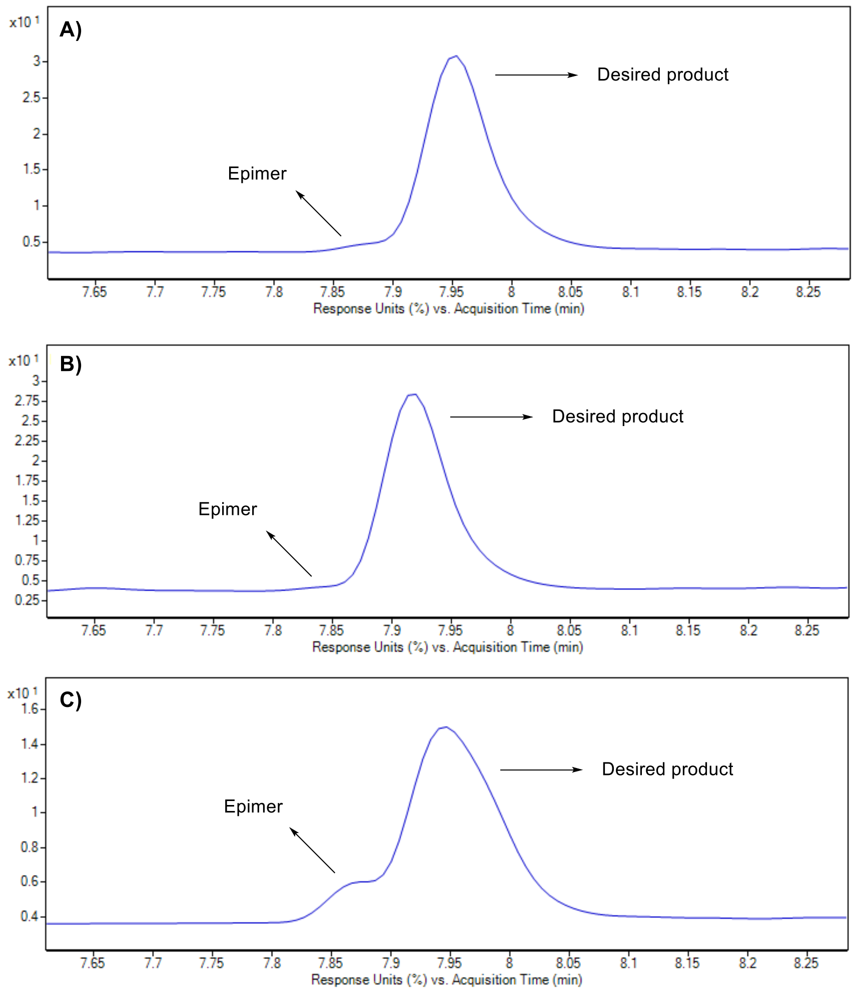

Zoomed C18 RP-HPLC-MS analysis of the reaction mixture of Fmoc-βAla-Val-Cit-OMe. A) Reaction with HATU as coupling reagent. B) Reaction with PyAOP as coupling reagent. C) Reaction with DEPBT as coupling reagent.

9 Chemicals, equipment and materials

Table S4: Chemicals, equipment and materials.

Product	Manufacturer
8-well Nunc Lab-Tek chambered coverglass (155411)	Thermo Fisher Scientific
8-well Nunc Lab-Tek II chambered coverglass (155409)	Thermo Fisher Scientific
A431	ATCC CRL-1555
AdvanceBio Peptide Map	Agilent Technologies
Agilent 1200 HPLC-System	Agilent Technologies
Äkta Ettan	GE Healthcare
Äkta Explorer	GE Healthcare
Buffer filtration system	Millipore
Centrifuge 5810R	Eppendorf
Centrifuge Eppendorf 5417R	Eppendorf
Centrifuge Eppendorf 5424	Eppendorf
ClCCA matrix	Sigma-Aldrich
Compact QTOF mass spectrometer	Bruker Daltonics
Cryotome	Leica CM1950
DBCO-PEG$_3$-VC-PAB-MMAE	MedChemExpress
ECL CCD camera LAS 3000	Fuji Photo Film GmbH
Fluorescence filter LAS 3000 FL-Y515	Fujifilm
French press	SLM Aminco
French Press cell	Aminco
HisTrap™ HP	GE Healthcare
HiTrapTM Phenyl HP	GE Healthcare
HiTrapTM Protein A HP	GE Healthcare
LaChrom HPLC system	Merck Hitachi
Lago optical imaging system	Spectral Instruments Imaging
LysoTracker Green DND 26	Invitrogen
LysoTracker Red DND-99	Invitrogen
MCF7	ATCC HTB-22
Microtiter plate reader tecan infinite, Tecan M200	Tecan
Microtiter plate reader Infinite M Plex	Tecan
Microtiter plate reader tecan Spark, Multimode Microplate Reader	Tecan
Milli-Q-POD water purification system	Millipore GmbH
NanoDrop2000c	Thermo Fisher Scientific
NMR spectrometer Bruker Avance III 500	Bruker Daltonics
NMR spectrometer Bruker DRX 500	Bruker Daltonics
PD-10 Desalting Column	GE Healthcare
Thermomixer 5436, 1.5 mL	Eppendorf
Thermomixer *comfort*	Eppendorf
UltrafleXtremeTM MALDI-ToF/ToF	Bruker Daltonics
Vivaspin® Turbo 15 Ultrafiltration Unit	Sartorius
Vortex Genie 2	New Brunswick Scientific
Vortex Genie K550-GE	New Brunswick Scientific
Vortex G-560	Scientific Industries Inc.

10 Nucleic acid sequence of DARPin dimer

Quality Assurance Documentation

invitrogen by Thermo Fisher Scientific

**ProjectID:**	2019AASCLP	**ConstructID:**	19ACAALP
**Gene Name:**	DARPin_Dimer	**Gene Size:**	1074 bp
**Designation:**	E.coli K12 DH10B™ T1R	**Vector Backbone:**	pMA-RQ (AmpR)
**Manufacturing Date:**	11 June 2019	**Quantity:**	~5 µg Plasmid DNA

Product Description: The synthetic gene DARPin_Dimer was assembled from synthetic oligonucleotides and/or PCR products. The fragment was inserted into pMA-RQ (AmpR). The plasmid DNA was purified from transformed bacteria and concentration determined by UV spectroscopy. The final construct was verified by sequencing. The sequence identity within the insertion sites was 100%. 5 µg of the plasmid preparation were vacuum dried for shipping.

Product Handling: The delivered DNA amounts are indicated on the individual tube labels. Centrifuge tubes prior to opening. Do not store vacuum dried DNA for a prolonged time. Add an appropriate amount of distilled water or 10 mM Tris-HCl (pH 8.5) and incubate for 1 hour at room temperature (optionally followed by an overnight incubation at 4°C). Resuspend DNA by gently pipetting up and down a couple of times. If not to be used immediately, resuspended DNA should be stored at -20°C or -80°C. Storing as aliquots helps to reduce unfavorable freeze-thaw cycles. We recommend sequence verification after each subcloning respectively transformation step.

Plasmid Map:

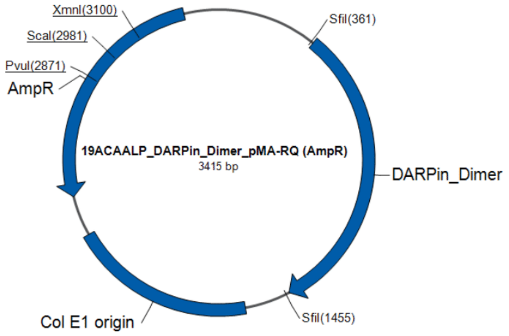

Data Handling: If you have subscribed to our convenient service to deliver all Quality Assurance Documentation (QAD) for your GeneArt orders electronically through Thermo Fisher Cloud, please follow this link to access your information: https://apps.thermofisher.com/apps/geneart-qa. Alternatively, you can scan the QR code.
If you have not subscribed to this service, the QAD can be found on the CD included in your shipment. The QAD includes a .gb file containing the full vector information, a .fas file for your insert sequence only and if applicable, your ordered raw sequencing data.

11 June 2019 - This quality confirmation was generated automatically and is valid without signature.
For research use only. Not for use in diagnostic procedures.

DARPin Dimer (DD2) nucleic acid sequence of commercially obtained plasmid

CACTATAGGGCGAATTGGCGGAAGGCCGTCAAGGCCGCAT**CATATG**GCAGGCGCAGTTGGTCTGTG

TACCCCGAGCCGTGCCGGTGATCTGGGTAAAAAACTGCTGGAAGCAGCACGTGCAGGTCAGGATGA

TGAAGTTCGTATTCTGATGGCAAATGGTGCAGATGTTAATGCAGATGATACCTGGGGTTGGACACCG

CTGCATCTGGCAGCATATCAGGGTCATCTGGAAATTGTTGAAGTGCTGCTGAAAAACGGTGCCGATG

TGAATGCCTATGATTATATTGGTTGGACCCCTCTGCACTTAGCAGCAGATGGCCATTTAGAAATCGT

GGAAGTTTTACTGAAAAATGGCGCAGACGTGAACGCAAGCGATTATATCGGTGATACCCCGTTACA

CCTGGCAGCACATAATGGCCACCTGGAAATCGTAGAGGTTCTGTTAAAACATGGTGCGGACGTGAA

TGCACAGGATAAATTTGGTAAAACCGCCTTCGATATCAGCATTGATAATGGCAATGAAGATCTGGC

CGAAATTCTGCAAGGTGGTGGTGGTAGCGGTGGTGGCGGTTCAGGTGGCGGTGGTTCTGGCGGTGGC

GGTAGCGATTTAGGCAAAAAACTGTTAGAGGCTGCGCGTGCTGGCCAGGATGACGAGGTGCGCATT

CTTATGGCCAACGGTGCTGACGTAAACGCCGATGACACATGGGGCTGGACGCCTTTACATTTAGCTG

CCTATCAGGGACACTTAGAGATTGTCGAGGTTCTGCTGAAGAATGGGGCTGATGTTAACGCGTATGA

TTACATCGGCTGGACTCCACTGCATCTTGCTGCCGATGGTCACCTTGAGATCGTTGAGGTATTACTTA

AGAATGGTGCGGATGTAAACGCCAGCGATTACATTGGCGATACGCCACTGCATTTAGCCGCTCATA

ATGGTCACTTAGAAATAGTCGAAGTCCTGTTAAAGCATGGCGCTGATGTAAATGCCCAAGATAAATT

CGGCAAGACGGCATTTGATATTAGCATCGATAACGGTAACGAGGACCTGGCAGAAATCCTGCAGCT

GTGCACCCCGTCACGTGGTGGTCATCATCACCATCATCATTAA**AAGCTT**CTGGGCCTCATGGGCCTT

CCGCTCACTGCCCGCTTTCCAG

Green: NdeI cleavage site; Purple: HindII cleavage site; underlined: DARPin Dimer (DD2)

11 Amino acid sequences

11.1 hFGE

ADLGSSMEFEANAPGPVPGERQLAHSKMVPIPAGVFTMGTDDPQIKQDGEAPARRVTIDAFYMDAYEVS

NTEFEKFVNSTGYLTEAEKFGDSFVFEGMLSEQVKTNIQQAVAAAPWWLPVKGANWRHPEGPDSTILHR

PDHPVLHVSWNDAVAYCTWAGKRLPTEAEWEYSCRGGLHNRLFPWGNKLQPKGQHYANIWQGEFPVT

NTGEDGFQGTAPVDAFPPNGYGLYNIVGNAWEWTSDWWTVHHSVEETLNPKGPPSGKDRVKKGGSYM

CHRSYCYRYRCAARSQNTPDSSASNLGFRCAADRLPTMDSGRGS**HHHHHHH**

11.2 MtFGE

MGSS**HHHHHH**SSGLCTPSRVPRGSHMLTELVDLPGGSFRMGSTRFYPEEAPIHTVTVRAFAVERHPVTNA

QFAEFVSATGYVTVAEQPLDPGLYPGVDAADLCPGAMVFCPTAGPVDLRDWRQWWDWVPGACWRHPF

GRDSDIADRAGHPVVQVAYPDAVAYARWAGRRLPTEAEWEYAARGGTTATYAWGDQEKPGGMLMAN

TWQGRFPYRNDGALGWVGTSPVGRFPANGFGLLDMIGNVWEWTTTEFYPHHRIDPPSTACCAPVKLATA ADPTISQTLKGGSHLCAPEYCHRYRPAARSPQSQDTATTHIGFRCVADPVSG

11.3 DARPin Monomer

MDLGKKLLEAARAGQDDEVRILMANGADVNADDTWGWTPLHLAAYQGHLEIVEVLLKNGADVNAYD YIGWTPLHLAADGHLEIVEVLLKNGADVNASDYIGDTPLHLAAHNGHLEIVEVLLKHGADVNAQDKFG KTAFDISIDNGNEDLAEILQL**CTPSR**GG**HHHHHH**

11.4 DARPin Dimer (with C-terminal CTPSR)

MDLGKKLLEAARAGQDDEVRILMANGADVNADDTWGWTPLHLAAYQGHLEIVEVLLKNGADVNAYD YIGWTPLHLAADGHLEIVEVLLKNGADVNASDYIGDTPLHLAAHNGHLEIVEVLLKHGADVNAQDKFG KTAFDISIDNGNEDLAEILQGGGGSGGGGSGGGGSGGGGSDLGKKLLEAARAGQDDEVRILMANGADVN ADDTWGWTPLHLAAYQGHLEIVEVLLKNGADVNAYDYIGWTPLHLAADGHLEIVEVLLKNGADVNAS DYIGDTPLHLAAHNGHLEIVEVLLKHGADVNAQDKFGKTAFDISIDNGNEDLAEILQL**CTPSR**GG**HHHH HH**

11.5 DARPin Dimer (with N- and C-terminal CTPSR)

MAGAVGL**CTPSR**AGDLGKKLLEAARAGQDDEVRILMANGADVNADDTWGWTPLHLAAYQGHLEIVE VLLKNGADVNAYDYIGWTPLHLAADGHLEIVEVLLKNGADVNASDYIGDTPLHLAAHNGHLEIVEVLLK HGADVNAQDKFGKTAFDISIDNGNEDLAEILQGGGGSGGGGSGGGGSGGGGSDLGKKLLEAARAGQDD EVRILMANGADVNADDTWGWTPLHLAAYQGHLEIVEVLLKNGADVNAYDYIGWTPLHLAADGHLEIV EVLLKNGADVNASDYIGDTPLHLAAHNGHLEIVEVLLKHGADVNAQDKFGKTAFDISIDNGNEDLAEIL QL**CTPSR**GG**HHHHHH**

11.6 DARPin Dimer (with C-terminal CTPSR)

MDLGKKLLEAARAGQDDEVRILMANGADVNADDTWGWTPLHLAAYQGHLEIVEVLLKNGADVNAYD YIGWTPLHLAADGHLEIVEVLLKNGADVNASDYIGDTPLHLAAHNGHLEIVEVLLKHGADVNAQDKFG KTAFDISIDNGNEDLAEILQGGGGSGGGGSGGGGSGGGGSDLGKKLLEAARAGQDDEVRILMANGADVN ADDTWGWTPLHLAAYQGHLEIVEVLLKNGADVNAYDYIGWTPLHLAADGHLEIVEVLLKNGADVNAS DYIGDTPLHLAAHNGHLEIVEVLLKHGADVNAQDKFGKTAFDISIDNGNEDLAEILQL**CTPSR**GG**HHHH HH**

11.7 DARPinFc

SDLGKKLLEAARAGQDDEVRILMANGADVNADDTWGWTPLHLAAYQGHLEIVEVLLKNGADVNAYDY IGWTPLHLAADGHLEIVEVLLKNGADVNASDYIGDTPLHLAAHNGHLEIVEVLLKHGADVNAQDKFGK

TAFDISIDNGNEDLAEILQGGSGGGSGDKTHTCPPCPAPELLGGPSVFLFPPKPKDTLMISRTPEVTCVVVD
VSHEDPEVKFNWYVDGVEVHNAKTKPREEQYNSTYRVVSVLTVLHQDWLNGKEYKCKVSNKALPAPIEK
TISKAKGQPREPQVYTLPPSRDELTKNQVSLTCLVKGFYPSDIAVEWESNGQPENNYKTTPPVLDSDGSFFL
YSKLTVDKSRWQQGNVFSCSVMHEALHNHYTQKSLSLSPGKDIGGSGGL**CTPSR**AA**HHHHHH**

11.8 scFv425 Fc

ASDYKDEVQLQQSGAELVKPGASVKLSCKASGYTFTSHWMHWVKQRAGQGLEWIGEFNPSNGRTNYNE
KFKSKATLTVDKSSSTAYMQLSSLTSEDSAVYYCASRDYDYDGRYFDYWGQGTTVTVSSGGGGSGGGGSG
GGGSDIELTQSPAIMSASPGEKVTMTCSASSSVTYMYWYQQKPGSSPRLLIYDTSNLASGVPVRFSGSGSGTS
YSLTISRMEAEDAATYYCQQWSSHIFTFGSGTELEIKREPKSCDKTHTCPPCPAPELLGGPSVFLFPPKPKDTL
MISRTPEVTCVVVDVSHEDPEVKFNWYVDGVEVHNAKTKPREEQYNSTYRVVSVLTVLHQDWLNGKEYK
CKVSNKALPAPIEKTISKAKGQPREPQVYTLPPSRDELTKNQVSLTCLVKGFYPSDIAVEWESNGQPENNYK
TTPPVLDSDGSFFLYSKLTVDKSRWQQGNVFSCSVMHEALHNHYTQKSLSLSPGKDGGEQSSTAGRAAFIT
GQGL**CTPSR**TG
